# Differential Privacy Preservation for Continuous Release of Real-Time Location Data

**DOI:** 10.3390/e26020138

**Published:** 2024-02-03

**Authors:** Lihui Mao, Zhengquan Xu

**Affiliations:** State Key Laboratory of Information Engineering in Surveying, Mapping and Remote Sensing, Wuhan University, Wuhan 430079, China; maolihui@whu.edu.cn

**Keywords:** location privacy preservation mechanism, differential privacy, geo indistinguishability, series indistinguishability, Correlated Laplace Mechanism

## Abstract

Continuous real-time location data is very important in the big data era, but the privacy issues involved is also a considerable topic. It is not only necessary to protect the location privacy at each release moment, but also have to consider the impact of data correlation. Correlated Laplace Mechanism (CLM) is a sophisticated method to implement differential privacy on correlated time series. This paper aims to solve the key problems of applying CLM in continuous location release. Based on the finding that the location increment is approximately stationary in many scenarios, a location correlation estimation method based on the location increment is proposed to solve the problem of nonstationary location data correlation estimation; an adaptive adjustment model for the CLM filter based on parameter quantization idea (QCLM) as well as its effective implementation named QCLM-Lowpass utilizing the lowpass spectral characteristics of location data series is proposed to solve the problem of output deviations due to the undesired transient response of the CLM filter in time-varying environments. Extensive simulations and real data experiments validate the effectiveness of the proposed approach and show that the privacy scheme based on QCLM-Lowpass can offer a better balance between the ability to resist correlation-based attacks and data availability.

## 1. Introduction

With the prevalence of intelligent terminal devices equipped with high-precision positioning capabilities, people can access location-based services (LBSs) whenever and wherever they want. As a result, large amounts of individuals’ location data are collected, stored, and analyzed, which has become an important resource for business analysis and academic research. However, location data is inherently sensitive and can be linked to data from other sources since space brings particular constraints [1,2], which enables an attacker to infer individual private information such as home address, company, lifestyle habits, etc. Privacy concerns hinder users’ willingness to share location data. Therefore, location privacy protection is one of the important issues in the big data era.

A variety of location-privacy-preserving mechanisms (LPPMs) have been proposed in the literature, and some works [2,3,4] provide a systematic review of them. Among the existing privacy definitions, differential privacy [5] based on the idea of indistinguishability in cryptography provides a rigorous mathematical proof of the privacy strength, which guarantees that the actual privacy strength is not affected by the attacker’s background knowledge, and thus is widely used in location-based services [6,7,8]. Early works on differential privacy assumed the presence of trusted third-party administrators, but as application environments have become more complex, many untrustworthy servers can also easily access large amounts of users’ data. To solve this problem, local differential privacy (LDP) [9] has been proposed. It perturbs the user’s data locally before sending the data to any entity. Only the data owner can fully access the original data, which provides stronger privacy protection. In the field of location privacy, geo-indistinguishability [10] extended the idea of indistinguishability to continuous geographic space, enabling local privacy protection for individual locations in snapshot publishing.

However, the privacy problem is more serious in scenarios of continuous location data publishing [2]. The continuously observed location data is usually correlated, which may make it difficult for differential privacy mechanisms to achieve the expected privacy-preserving effect [11,12]. Some existing works for correlated location data publishing mainly take advantage of a model to describe the correlation between locations, and thereby adapt the location perturbation approach. Jiang et al. [13] considered time-space constraints between locations according to a basic kinematics model, and used an exponential mechanism to generate azimuths and distances which are more consistent with the motion pattern, thus avoiding irrationally perturbed locations. Chatzikokolakis et al. [14] used a prediction function to characterize the correlation between locations, and privately determined whether to allocate a privacy budget to generate noise for the current location based on the error of the prediction. Instead, Al-Dhubhani et al. [15] adaptively adjusted the privacy budget at each moment based on the level of linear prediction error. In addition, Xiao et al. [16] constructed the “δ-location set” based on a Markov model to account for the temporal correlations in location data and proposed a planar isotropic mechanism (PIM) for location perturbation. Based on this work, Xiong et al. [17] improved data usability by applying a generalized randomized response mechanism (GRR) to the “δ-location set” for privacy protection. Unfortunately, these works lacked an exhaustive explanation for why data correlation would lead to a reduction in the privacy strength of differential privacy mechanisms.

Wang and Xu [18] argued that the primary cause of the reduction in privacy strength is the discrepancy in data correlation between the noise and original series. This can be used by an attacker to launch a correlation-distinguishability attack (CDA) [12], such as Weiner filtering, to filter out some of the perturbation noise and thus remove some of the privacy effect. To remedy this problem, the work [18] proposed the notion of series-indistinguishability to guarantee that the correlation between the noise and original series is indistinguishable, and designed the Correlated Laplace Mechanism (CLM) to generate a correlated Laplace noise series to satisfy series-indistinguishability, which provided an effective differential privacy-preserving scheme for correlated time-series data publication. Naturally, it is desirable to apply CLM to protect privacy in continuous location data release.

However, there are some challenges in the implementation of CLM, which mainly include the following two aspects:

1. How to accurately estimate the data correlation. A prerequisite for the effective application of CLM is that the autocorrelation function of the data series is known or can be accurately estimated. However, the time series consisting of continuously observed location data is usually non-stationary, and its autocorrelation function changes over time, which makes it difficult to obtain accurate estimates based on time averaging.
2. How to make CLM dynamically track the time-varying data correlation. CLM based on the filter approach is performed by setting the filter parameters so that the steady-state response satisfies the given autocorrelation function. To track the time-varying data correlation, the CLM filter should be dynamically adjusted, which may cause the undesired transient response to be non-negligible, resulting in a deviation of the actual output from the target.

The above challenges make CLM unsuitable for continuous real-time location data release. This paper presents practical solutions to these challenges. First, the stationarity of the location data series is analyzed, and results imply that the location increment is approximately stationary in many scenarios, from which a location data correlation estimation method using the location increment is derived. Second, an adaptive adjustment model of the CLM filter based on parameter quantization, called Quantized Correlated Laplace Mechanism (QCLM), is proposed, and based on the lowpass spectral characteristics of the location data series, an effective implementation named QCLM-Lowpass is designed. Our main contributions are summarized as follows:

- A location data correlation estimation method based on the location increment is proposed, in which the problem of correlation estimation of a nonstationary location data series is converted into the problem of estimating real location increments. Thereby, the correlation of nonstationary location data can be accurately estimated in many practical scenarios.
- An adaptive adjustment model of the CLM filter (QCLM) and its effective implementation (QCLM-Lowpass) are proposed. The CLM filter is adjusted only at the necessary moments to reduce the generation of undesired transient responses, thus producing an output that satisfies series-indistinguishability as much as possible in time-varying environments.
- Extensive simulations and real data experiments validate the effectiveness of the proposed approaches, and the results show that the privacy scheme based on QCLM-Lowpass outperforms the other schemes compared in terms of data usability and the ability to resist the correlation-based attack.

The remainder of the paper is organized as follows: Section 2 introduces the continuous real-time location data release model and relevant privacy theories and gives the privacy goals in this paper. The Dynamic Correlated Laplace Mechanism (DCLM) and problem statement are presented in Section 3. The location data correlation estimation method and QCLM are proposed in Section 4. We report the experimental evaluation in Section 5 and give the conclusion in Section 6.

## 2. Preliminaries

In this section, the continuous real-time location data release model is introduced, and then several relevant privacy theories are reviewed, followed by the privacy-preserving goals in this paper.

### 2.1. Continuous Real-Time Location Data Release Model

**Figure 1:**
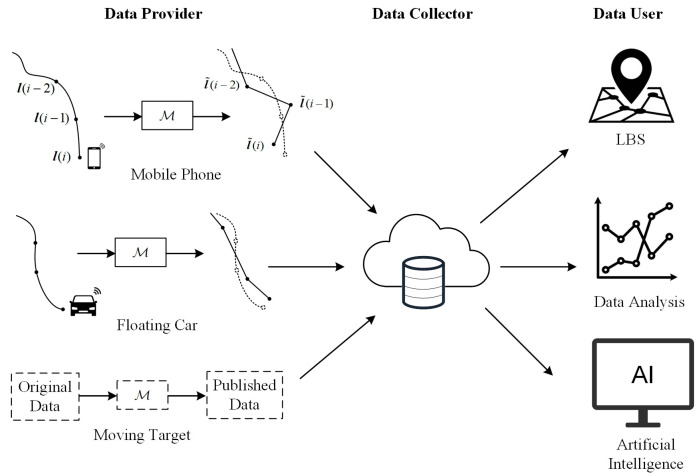
Location data ecosystem based on local privacy protection.

Before introducing the continuous location data release model, the location data ecosystem is presented in Figure 1. There are three important roles: the data provider, the data collector, and the data user, each with different demands. Data users expect accurate location data for application analysis, while data providers are reluctant to provide raw data for privacy reasons. The role of privacy protection is to balance these requirements, protecting user privacy while ensuring the usability of published data. Therefore, privacy protection is vital to the healthy and sustainable development of the location data ecosystem.

**Figure 2:**
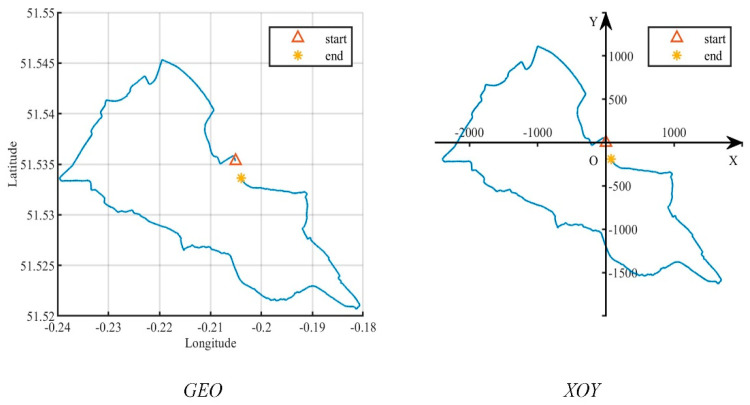
Two coordinate systems.

All spatial data is created in a coordinate system. This paper involves two coordinate systems, denoted as *GEO* and *XOY*. In *GEO*, a location record is expressed as loc(t)={ID,t,<lon(t),lat(t),alt(t)>}, where *ID* denotes the user identity, and t denotes the time stamp, lon(t),lat(t),alt(t) indicate longitude, latitude, and altitude information, respectively. Given that the user usually moves within a limited area where altitude changes can be ignored, a Cartesian coordinate system, *XOY*, is introduced. Specifically, the coordinate $<lon(t_{0}),lat(t_{0})>$ at the certain moment $t_{0}$ is used as the origin, *O*, and *XOY* can be constructed with the east direction as the positive direction of *X*-axis, the north direction as the positive direction of *Y*-axis, and the scale in meters. Thereby, a location at time t can be expressed by the vector $l(t)={\left[x(t),y(t)\right]}^{T}$, where the superscript T denotes the matrix transpose and x(t),y(t) are the coordinates on the *X* and *Y* axes, respectively. An example for such two coordinate systems is shown in Figure 2.

In practice, many applications require the user to continuously share real-time location data. In a typical scenario, the user releases location data with a fixed time interval, Δt, starting from a certain moment, $t_{0}$. For convenience, the *i*-th release time is $t_{i}=t_{0}+i\times \Delta t$, and the corresponding location data is denoted as $l(i)={\left[x(i),y(i)\right]}^{T}$. In the local privacy model shown in Figure 1, the user does not directly release the original location l(i) but releases a perturbed location, $\tilde{l}(i)={\left[\tilde{x}(i),\tilde{y}(i)\right]}^{T}$, which is the output of the privacy mechanism M:

(1) $$\tilde{l}(i)=M[l(i)]=l(i)+n(i)$$

where $n(i)={\left[n_{X}(i),n_{Y}(i)\right]}^{T}$ is the noise vector and $n_{X}(i),n_{Y}(i)$ are the noise on the *X* and *Y* directions, respectively.

Suppose that there have been I releases until now, then the data series formed by original locations can be denoted as $L(I)=[X(I),Y(I)]={\left[l(0),\cdots ,l(I)\right]}^{T}$, where the column vectors $X(I)={\left[x(0),\cdots ,x(I)\right]}^{T}$ and $Y(I)={\left[y(0),\cdots ,y(I)\right]}^{T}$ are the data series in the *X* and *Y* directions. Similarly, the noise series and the perturbed location series are denoted as $N(I)=[N_{X}(I),N_{Y}(I)]={\left[n(0),\cdots ,n(I)\right]}^{T}$ and $\tilde{L}(I)=[\tilde{X}(I),\tilde{Y}(I)]={\left[\tilde{l}(0),\cdots ,\tilde{l}(I)\right]}^{T}$ respectively. The major notations in this paper are summarized in Table 1.

To protect privacy, it should be guaranteed that the attacker cannot accurately infer true location information based on the published data, including the following demands,

1. The attacker cannot accurately infer the original location l(i) from $\tilde{l}(i)$ at each moment.
2. The attacker cannot accurately extrapolate the true location l(i) based on the historically published series $\tilde{L}(I)$.

### 2.2. Privacy Theories

For this privacy problem, we first review several important privacy theories.

#### 2.2.1. Differential Privacy

Differential privacy [5] is based on the concept of indistinguishability in cryptology and is a state-of-the-art privacy preservation model. It ensures that the output of a private algorithm is not strongly dependent on any one record in the input dataset. Thus, differential privacy provides strict privacy protection, even if the attacker knows all information except the target record. Its formal definition is given below.

**Definition** **1** (differential privacy [5])**.** *Considering two neighboring datasets, $D,D^{\prime }$, which have the same cardinality but differ in only one record, a random perturbation mechanism, M, satisfies (ε,δ)-differential privacy if for all possible outcomes O⊆Range(M) and for any pair of $D,D^{\prime }$:*

(2) $$Pr[M(D)\in O]\leq e^{\varepsilon }\cdot Pr[M(D^{\prime })\in O]+\delta$$

*where Range(M) denotes the value range of M, and Pr(⋅) denotes the probability. If δ=0, we say that M is ε-differentially private. The parameter ε is considered as the privacy budget which indicates the privacy strength. A small ε is associated with better privacy.*

A popular ε-differentially private algorithm for numeric queries is the Laplace mechanism. The definition of the Laplace mechanism is given below.

**Definition** **2** (Laplace mechanism [19])**.** *Let Lap(λ) denote the Laplace distribution with mean 0 and scale λ. For any numeric-valued function $f:D\to {\mathbb{R}}^{k}$, the Laplace mechanism $M_{L}$ is defined as follows:*

(3) $$M_{L}(D,f(\cdot ),\varepsilon )=f(D)+<N_{1},\cdots ,N_{k}>$$

*where $<N_{1},\cdots ,N_{k}>$ are i.i.d. random variables drawn from Lap(Δf/ε). The sensitivity function Δf is the maximum effect a single record has on the result of f(⋅):*

(4) $$\Delta f=\underset{D,D^{\prime }}{\max}{\left\|f(D)-f(D^{\prime })\right\|}_{1}$$

#### 2.2.2. Geo-Indistinguishability

The idea of indistinguishability is the basis of differential privacy, geo-indistinguishability [10] extends it to the continuous geographic space, which can be formally defined as follows.

**Definition** **3** (geo-indistinguishability [10])**.** *A mechanism M satisfies ε-geo-indistinguishability if for any two locations $l,l^{\prime }$ in the protection region and for any output location $\tilde{l}\in Range(M)$:*

(5) $$Pr[M(l)=\tilde{l}]\leq e^{\varepsilon \cdot d_{L}(l,l^{\prime })}\cdot Pr[M(l^{\prime })=\tilde{l}]$$

*where $d_{L}(\cdot ,\cdot )$ denote the distance between two locations, such as Euclidean distance.*

Geo-indistinguishability ensures that it is difficult for an attacker to distinguish the true location from its surroundings according to the perturbed location, and thus provides privacy protection for the single location. Its privacy strength is controlled by the parameter ε.

#### 2.2.3. Series-Indistinguishability

Continuously observed location data will constitute a correlated time series, which is vulnerable to correlation-based attacks. To solve this problem, series-indistinguishability [18] has been proposed to complement differential privacy preservation on correlated time-series data. Its formal definition is given as follows.

**Definition** **4** (series-indistinguishability [18])**.** *Let $X,\tilde{X}$ denote the original and perturbed data series. If the autocorrelation functions of them, $R_{X}(\tau )$ and $R_{\tilde{X}}(\tau )$, satisfy the following:*

(6) $$R_{\tilde{X}}(\tau )/R_{\tilde{X}}(0)=R_{X}(\tau )/R_{X}(0)$$

*then $X,\tilde{X}$ are series-indistinguishable to an adversary, where τ∈ℤ denotes the lag of the auto-correlation function.*

In practice, however, it is difficult to achieve absolute series-indistinguishability due to various factors such as estimation errors. A more robust extension with noise tolerant interval [−υ,υ] was presented, which requires the autocorrelation functions to satisfy the following condition:

(7) $$-\upsilon \leq \frac{R_{\tilde{X}}(\tau )}{R_{\tilde{X}}(0)}/\frac{R_{X}(\tau )}{R_{X}(0)}\leq \upsilon$$

Series-indistinguishability makes it difficult for an attacker to filter out some of the perturbation noise by exploiting the data correlation, so that the effective privacy protection of the correlated time series can be achieved without increasing the noise intensity.

#### 2.2.4. Correlated Laplace Mechanism

To achieve series-indistinguishability, the privacy mechanism M needs to generate a noise series whose autocorrelation function is consistent with that of the original data series. For this purpose, CLM [18] was proposed to generate a correlated Laplace noise series with the given autocorrelation function. It includes the following two steps:

1. Generate four Gaussian distributed random numbers independently, $g_{m}^{\prime }(i)\sim N(0,\sigma_{G^{\prime }}^{2})$, m=1, 2, 3, 4, where i∈ℕ denotes the timestamp. Thereby, four Gaussian noise series are generated over time, denoted as $G_{m}^{\prime },m=1,\;2,\;3,\;4$. Each of them satisfies the same autocorrelation function $R_{G^{\prime }}(\tau )$, but are independent from each other.
2. Generate the Laplace distributed random number $n(i)\sim Lap(2\sigma_{G^{\prime }}^{2})$, which can be calculated as follows:
  (8) $$n(i)={\left[g_{1}^{\prime }(i)\right]}^{2}+{\left[g_{2}^{\prime }(i)\right]}^{2}-{\left[g_{3}^{\prime }(i)\right]}^{2}-{\left[g_{4}^{\prime }(i)\right]}^{2}$$
  Thereby, the correlated Laplace noise series can be generated over time, where the autocorrelation function of the Laplace and Gaussian noise series, $R_{N}(\tau )$ and $R_{G^{\prime }}(\tau )$, satisfy the following equation:
  (9) $$R_{N}(\tau )=8{\left[R_{G^{\prime }}(\tau )\right]}^{2}$$

**Figure 3:**
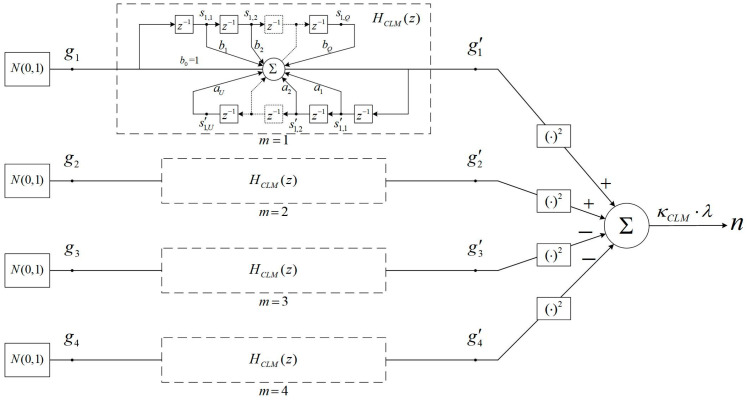
The process of CLM.

Figure 3 illustrates the generation of the correlated Laplace noise in CLM at a certain moment. There are four independently operating filters. They have the same parameters but different inputs and outputs. The filter used to generate the correlated Gaussian noise series with the input of Gaussian white noise is known as the CLM filter. It can be characterized by the following system function:

(10) $$H_{CLM}(z)=\frac{\sum_{q=0}^{Q}b_{q}z^{-q}}{\sum_{u=0}^{U}a_{u}z^{-u}}$$

where the complex variable $z=e^{\sigma +j\omega }$, and Q,U denote the order of zeros and poles, respectively. Let $a_{0}=b_{0}=1$ by default and let $A_{CLM}=[a_{1},\cdots ,a_{U}]$, $B_{CLM}=[b_{1},\cdots ,b_{Q}]$ denote the parameter vectors. In addition, there is a gain coefficient $\kappa_{CLM}$ in the calculation of the Laplace noise n~Lap(λ), which can be calculated as follows:

(11) $$\kappa_{CLM}=\pi /\int_{-\pi }^{\pi }{\left\|H_{CLM}(z){|}_{z=e^{j\omega }}\right\|}_{2}^{2}d\omega$$

To clearly describe the dynamic adjustment problem of the CLM filter, this paper considers CLM as a program object, and redescribes its implementation from a programming perspective. Table 2 lists the main attributes and methods of the CLM object.

At the beginning of the application, the method Initialization is called to assign initial values to the parameters and clear the state of filters. The methods UpdateParam and UpdateGainCoeff are used to update the parameters when it is necessary. During each iteration, the method LaplaceGeneration is called to generate the Laplace noise, where the output of GaussianGenerator is used as the input of the CLM filter.

This paper focuses on the generation of correlated Laplace noise, and the specific steps of the method LaplaceGeneration are given in Algorithm 1.

**Algorithm** **1.** CLM: LaplaceGeneration( )1Generate four i.i.d random numbers,   $\left\{g_{1},g_{2},g_{3},g_{4}\}=GaussianGenerator(\;\right)$Set $G={\left[g_{1},g_{2},g_{3},g_{4}\right]}^{T}$2Calculate four correlated Gaussian noise,

$G^{\prime }=-S^{\prime }A^{T}+G+SB^{T}$

3Update the input state matrix *S*,     $\begin{array}{cc} s_{m,k+1}\leftarrow s_{m,k} & k=1,\cdots ,Q-1,m=1,2,3,4\\ s_{m,1}\leftarrow g_{m} & m=1,2,3,4 \end{array}$4Update the output state matrix *S*′,     $\begin{array}{cc} s_{m,k+1}^{\prime }\leftarrow s_{m,k}^{\prime } & k=1,\cdots ,U-1,m=1,2,3,4\\ s_{m,1}^{\prime }\leftarrow g_{m}^{\prime } & m=1,2,3,4 \end{array}$5Calculate the noise n~Lap(λ),     $n=[{\left(g_{1}^{\prime }\right)}^{2}+{\left(g_{2}^{\prime }\right)}^{2}-{\left(g_{3}^{\prime }\right)}^{2}-{\left(g_{4}^{\prime }\right)}^{2}]\cdot \kappa \cdot \lambda$6**return** *n*

In line 1, the method GaussianGenerator is called to generate i.i.d random numbers $g_{1},g_{2},g_{3},g_{4}\sim N(0,1)$, which are used as the inputs of the four filters. In lines 2–4, the outputs $g_{1}^{\prime },g_{2}^{\prime },g_{3}^{\prime },g_{4}^{\prime }$ are calculated and the states of filters are updated. In lines 5–6, the noise n~Lap(λ) is generated and returned.

### 2.3. Privacy Protection Goals

Based on the above privacy theories, the privacy requirements presented in Section 2.1 can be expressed as follows,

1. At each release moment, if the privacy mechanism M satisfies ε-geo-indistinguishability as defined in Equation (12), then the attacker cannot easily and accurately infer the original location l(i) from the published location $\tilde{l}(i)$:
  (12) $$\frac{Pr\{M[l(i)]=\tilde{l}(i)\}}{Pr\{M[l^{\prime }(i)]=\tilde{l}(i)\}}\leq \exp\{\varepsilon \cdot d_{E}[l(i),l^{\prime }(i)]\}$$
  where $l^{\prime }(i)={\left[x^{\prime }(i),y^{\prime }(i)\right]}^{T}$ is a location near l(i) with a Euclidean distance $d_{E}[l(i),l^{\prime }(i)]$.
2. As for the original and perturbed location series, $L(I),\tilde{L}(I)$, if they are series-indistinguishable, then the attacker cannot easily use data correlation to accurately infer l(i) from $\tilde{L}(I)$. This requires that their autocorrelation function matrices, $R_{L}(i,i-\tau ),R_{\tilde{L}}(i,i-\tau )$, satisfy the following condition:
  (13) $$R_{L}(i,i-\tau )⊘R_{L}(i,i)=R_{\tilde{L}}(i,i-\tau )⊘R_{\tilde{L}}(i,i)$$
  where ⊘ denotes the Hadamard division (element-wise division), and $R_{L}(i,i-\tau ),R_{\tilde{L}}(i,i-\tau )$ are defined as follows:
  (14) $$R_{L}(i,i-\tau )=E[l(i)l^{T}(i-\tau )]=\left[\begin{array}{cc} R_{XX}(i,i-\tau ) & R_{XY}(i,i-\tau )\\ R_{YX}(i,i-\tau ) & R_{YY}(i,i-\tau ) \end{array}\right]$$
  (15) $$R_{\tilde{L}}(i,i-\tau )=E[\tilde{l}(i){\tilde{l}}^{T}(i-\tau )]=\left[\begin{array}{cc} R_{\tilde{X}\tilde{X}}(i,i-\tau ) & R_{\tilde{X}\tilde{Y}}(i,i-\tau )\\ R_{\tilde{Y}\tilde{X}}(i,i-\tau ) & R_{\tilde{Y}\tilde{Y}}(i,i-\tau ) \end{array}\right]$$

Naturally, it is considered that CLM can be applied to achieve the above privacy goals, but this will face some challenges in the practical implementation.

## 3. Problems of Dynamic Correlated Laplace Mechanism

For streaming data publishing, the Dynamic Correlated Laplace Mechanism is presented, and then its practical problems in continuous location data release are described.

### 3.1. Dynamic Correlated Laplace Mechanism

In the continuous location data release application, location data is dynamically generated and its correlation changes over time. To achieve series-indistinguishability, the CLM filter must be dynamically adjusted to track the time-varying data correlation. This scheme is called Dynamic Correlated Laplace Mechanism (DCLM).

Let us first consider the one-dimensional data series and take X(I) as an example, DCLM can be implemented with a sliding window model. At the moment $t_{i}$, the data in the window with size $M_{L}$, denoted as $X_{W}(i)={\left[x(i-M_{L}+1),\cdots ,x(i)\right]}^{T}$, can be used to estimate the autocorrelation function vector $R_{X}(i)={\left[R_{XX}(i,i),\cdots ,R_{XX}(i,i-ϒ)\right]}^{T}$, where $R_{XX}(i,i-\tau )=E[x(i)\cdot x(i-\tau )]$, τ=0,⋯,ϒ, and ϒ is the maximum lag. Then, the CLM filter is adjusted so that the autocorrelation function of the Laplace noise series can be matched to $R_{X}(i)$.

Similarly, this paper considers DCLM as a program object in which CLM is a member. The attributes of DCLM include the CLM filter’s parameter vectors, B,A, and the adjustment step $\mu_{DCLM}$. In addition, there are two main methods in DCLM: one is used to initialize the attributes as well as CLM, and the other is used to dynamically adjust the CLM filter to generate Laplace noise, denoted as Iteration, which is described in Algorithm 2.

**Algorithm** **2.** DCLM: Iteration**Input**: the autocorrelation function vector R**Output**: the Laplace noise n1Calculate the CLM filter’s estimated parameter vector $\hat{B},\hat{A}$ according to R;2Update the CLM filter’s parameter vector,

$\begin{array}{c} B=B+(\hat{B}-B)\cdot \mu_{DCLM}\\ A=A+(\hat{A}-A)\cdot \mu_{DCLM} \end{array}$

3Calculate the gain coefficient κ according to B,A;4Update the parameters of the CLM filter

$\begin{array}{l} CLM.UpdateParam(B,A)\\ CLM.UpdateGainCoeff(\kappa ) \end{array}$

5Generate the Laplace noise n=CLM.LaplaceGeneration( );6**return** *n*;

In line 1, the autocorrelation function vector is used to compute the estimated parameter vectors of the CLM filter, which can be referred to the work [18]. Lines 2–3 update the parameter vectors and the gain coefficient. Lines 4–6 call the methods of the CLM object to adjust the CLM filter and to generate the Laplace noise to return.

From the above steps, it can be found that DCLM can be effectively applied when X(I) satisfies stationary or short-time stationary, in which case $R_{X}(i)$ can be accurately estimated and $\mu_{DCLM}$ can be smaller to ensure that the CLM filter’s output is as expected. However, there are some challenges in implementing DCLM in the continuous location data release application.

### 3.2. Problems of DCLM in Continuous Location Data Release

In fact, location data series usually do not satisfy the stationary conditions, which makes it difficult to accurately estimate the autocorrelation function. In addition, the dynamic adjustment of the CLM filter causes the unwanted transient response. This results in a deviation between the actual output and the desired output.

#### 3.2.1. Correlation Estimation

As for the dynamically generated location data series L(I), the data in the window with size $M_{L}$, denoted as $L_{W}(i)={\left[l(i-M_{L}+1),\cdots ,l(i)\right]}^{T}$, is used to calculate the estimate of autocorrelation function matrix, ${\hat{R}}_{L}(i,i-\tau )$,

(16) $${\hat{R}}_{L}(i,i-\tau )=\frac{1}{M_{L}}\sum_{k=0}^{M_{L}-\tau -1}l(i-k)l^{T}(i-\tau -k)$$

From the above equation, the estimation accuracy depends on the stationarity of L(I). If the following stationary conditions are satisfied, the window size $M_{L}$ can be larger to ensure the accurate estimation,

(17) E[l(i)]=E[l(i−k)]

(18) $$R_{L}(i,i-\tau )=R_{L}(i-k,i-k-\tau )$$

where k∈ℤ. However, this requires the user must stay at a certain location, which results in an excessively limited application scenario.

**Figure 4:**
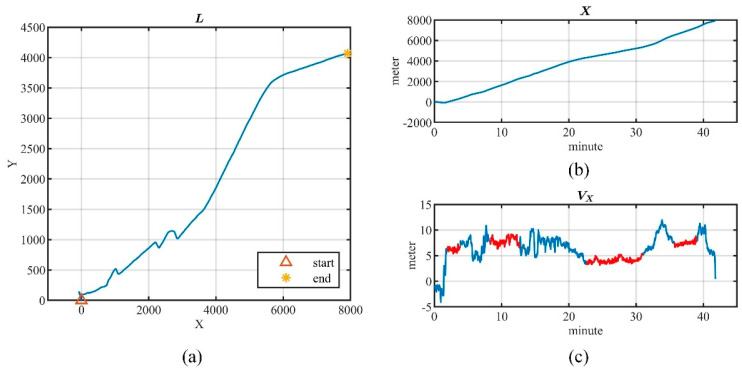
Real location series. (**a**) Real location series. (**b**) Location data series on X direction. (**c**) Location increment series on X direction.

A real location data series is shown in Figure 4a, and Figure 4b presents the data series X(I) on the *X* direction. As the user moves, X(I) shows a corresponding trend and thus no longer satisfies the stationary conditions. Therefore, it is difficult to accurately estimate the autocorrelation function directly from the original location data series.

#### 3.2.2. Transient Response in Dynamic Adjustment

Based on the knowledge of filter theory, the actual output of the CLM filter, $R_{CLM}(i)$, consists of two parts: the steady-state response, $R_{S}(i)$, and the transient response, $R_{T}(i)$:

(19) $$R_{CLM}(i)=R_{S}(i)+R_{T}(i)$$

where i∈ℕ is the timestamp. The former is determined by the input and the filter parameters $B_{CLM},A_{CLM}$, while the latter is determined not only by these two factors but also by the initial state of the filter. If $B_{CLM},A_{CLM}$ remain unchanged, then $R_{T}(i)$ will decays over time and eventually only $R_{S}(i)$ will remains. Therefore, to ensure the validity of the final result, $B_{CLM},A_{CLM}$ are adjusted to allow $R_{S}(i)$ to satisfy the given correlation, where $R_{T}(i)$ is the undesired part.

**Figure 5:**
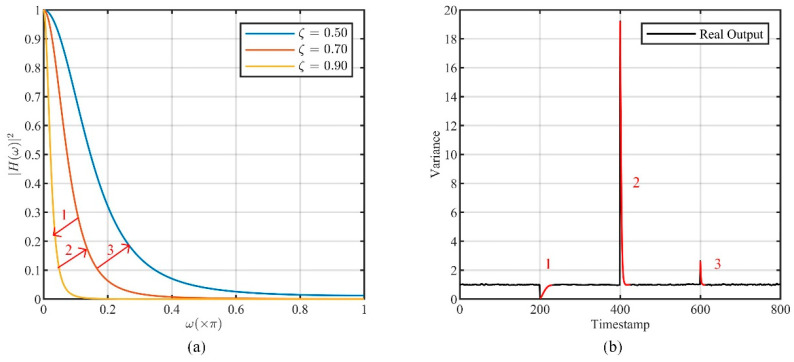
Transition phase after adjustment. (**a**) Changes in the normalized power spectrum of the filter during the adjustment process. (**b**) The variance of the actual output at each moment normalized by the steady-state response.

However, when $B_{CLM},A_{CLM}$ are changed, the previous steady state will be destroyed, and a new undesired transient response will be generated. This implies that a transition phase is required for the filter to return to steady state. Here, a second-order all-pole filter is taken as an example to illustrate this transition phase, its system function is as follows:

(20) $$H_{AP}(z)=\frac{1}{{\left(1-\zeta z^{-1}\right)}^{2}}$$

where ζ denotes the second-order pole, and we allow the Gaussian white noise G~N(0,1) as the input. Figure 5a shows the change of the filter’s normalized power spectrum in three adjustments, and Figure 5b presents the output variance over time, where the results are normalized according to the corresponding steady-state response.

As shown in Figure 5b, the filter’s adjustment produces the new transient response highlighted in red, which makes the actual results deviate from expectations. It takes some time for the filter to return to the steady state. Therefore, the undesired transient response needs to be suppressed in the implementation of DCLM.

## 4. Methodology

For the aforementioned practical problems, our solution idea includes the following two aspects:

1. Location data correlation estimation based on the location increment. The autocorrelation function of the location data is expressed in terms of true location increments. Thus, if the location increment series satisfies stationary conditions, the location data correlation can be calculated indirectly using the estimated location increments.
2. Quantized Correlated Laplace Mechanism (QCLM). On the one hand, the CLM filter should remain unchanged to suppress the transient response; on the other hand, it needs to be adjusted to track the time-varying data correlation. To balance these two requirements, this paper proposes an adaptive adjustment method based on parameter quantization. It adjusts the CLM filter only at the necessary moments, so that series-indistinguishability can be satisfied as much as possible.

### 4.1. Location Data Correlation Estimation Based on The Location Increment

In practice, it is desirable to find a more stationary intermediate variable to compute the autocorrelation function estimate for the non-stationary location data series. As illustrated in Figure 4c, the increments of X(I) show approximate stationarity in the time segments highlighted in red. Our analysis reveals that the location series with approximately stationary increments account for more than 27% of the real dataset (details are given in Section 5.1.3). Moreover, the location series and its increments can be mutually transformed with the given starting point. Therefore, we employ location increments to compute the autocorrelation function estimate for the location data.

For the locations l(i−1), l(i), their increment is denoted as $v(i)=l(i)-l(i-1)={\left[v_{X}(i),v_{Y}(i)\right]}^{T}$, and then the increment series of L(I) is denoted as $V(I)=[V_{X}(I),V_{Y}(I)]={\left[v(1),\cdots ,v(I)\right]}^{T}$, where $V_{X}(I)$ and $V_{Y}(I)$ are the increments in the *X* and *Y* directions. Similarly, stationarity require that E[v(i)] is constant over time, which means that the user remains at rest or in uniform linear motion, covering a wider range of scenarios.

Here, we describe the location data correlation estimation method based on location increments. To eliminate the effect of the coordinate origin, the location data correlation is characterized by the autocorrelation function matrix ${\tilde{R}}_{L}(i,i-\tau )$ after removing the center of $L_{W}(i)$:

(21) $${\tilde{R}}_{L}(i,i-\tau )=E\{[l(i)-O(i)]{\left[l(i-\tau )-O(i)\right]}^{T}\}$$

where the center O(i) is defined as follows:

(22) $$O(i)=\frac{1}{M_{L}}E[\sum_{k=0}^{M_{L}-1}l(i-k)]$$

In fact, l(i) is composed of two parts: the true value $l_{tr}(i)=E[l(i)]$ and the error $l_{er}(i)$, where tr,er denote the truth and error, respectively. In this paper, it is considered that the error series $L_{er}(I)={\left[l_{er}(0),\cdots ,l_{er}(I)\right]}^{T}$ is a zero-mean white noise, then Equation (21) can be further expressed as follows:

(23) $${\tilde{R}}_{L}(i,i-\tau )=[l_{tr}(i)-O_{tr}(i)]{\left[l_{tr}(i-\tau )-O_{tr}(i)\right]}^{T}$$

where $O_{tr}(i)=\sum_{k=0}^{M_{L}-1}l_{tr}(i-k)/M_{L}$. Furthermore, if $l(i-M_{L})$ is considered as the observation origin, then $l_{tr}(i-k)$ can be expressed as follows:

(24) $$l_{tr}(i-k)=l_{tr}(i-M_{L})+\sum_{m=k}^{M_{L}-1}v_{tr}(i-m)$$

where the true increment $v_{tr}(i)=l_{tr}(i)-l_{tr}(i-1)$. By substituting Equation (24) into Equation (23), the following equation can be obtained:

(25) $${\tilde{R}}_{L}(i,i-\tau )=[\sum_{m=0}^{M_{L}-1}v_{tr}(i-m)-O_{V}(i)]{\left[\sum_{m=\tau }^{M_{L}-1}v_{tr}(i-m)-O_{V}(i)\right]}^{T}$$

where $O_{V}(i)=\sum_{m=0}^{M_{L}-1}\left(m+1)\cdot v_{tr}(i-m\right)/M_{L}$.

By the above steps, the autocorrelation function of the location data is expressed in terms of true location increments. When V(I) satisfies the stationary conditions, the true location increments $v_{tr}(i)$ can be accurately estimated, and thus the location correlation ${\tilde{R}}_{L}(i,i-\tau )$ can be calculated according to Equation (25).

### 4.2. Quantized Correlated Laplace Mechanism

As for the dynamic adjustment of the CLM filter, an adaptive adjustment based on parameter quantization is proposed to achieve a balance between the suppression of transient response and the dynamic tracking of the time-varying correlations.

#### 4.2.1. Adaptive Adjustment Based on Parameter Quantization

**Figure 6:**
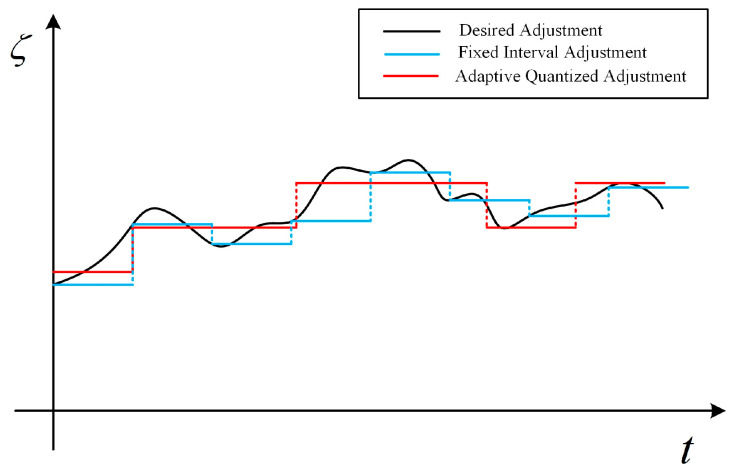
Different parameter adjustment schemes.

To suppress the transient response, the CLM filter should be kept unchanged to reach the steady state. A simple strategy is to control the time interval for parameter adjustment. For example, consider the filter with the system function given in Equation (20). As shown by the results highlighted in blue in Figure 6, the filter is adjusted at the set time interval. However, this method faces the following two problems,

1. It is difficult to determine the appropriate adjustment time. The CLM filter needs to be adjusted in time when the correlation of the output noise series does not match that of the original data. However, the time-varying data correlation is unpredictable, which makes it difficult to set the adjustment time interval in advance.
2. It is difficult to compute the appropriate parameters of the CLM filter. To reduce the effects of estimation errors or transient changes of data, it is necessary to determine the final adjustment based on the correlation estimate over a period of time. However, the computation of the filter parameters is non-linear, which makes it difficult to solve for the optimized results.

To this end, an adaptive adjustment strategy is desired. The basic idea is to constantly perceive the difference between the output of CLM and the target data series in terms of data correlation, and the CLM filter is only adjusted when the difference exceeds a certain threshold. As shown by the results highlighted in red in Figure 6, it adjusts the filter only when necessary, ensuring that the filter remains as unchanged as possible.

In this paper, we consider an adaptive adjustment scheme of the CLM filter based on parameter quantization, called Quantized Correlated Laplace Mechanism (QCLM). Let $H_{CLM}=[B_{CLM},A_{CLM}]$ denote the CLM filter’s parameter vector, and H denote the space including all possible value of $H_{CLM}$. In QCLM, H is divided into QNum mutually exclusive subspaces $H_{1},\cdots ,H_{QNum}$, which are mapped as different vectors $H_{1},\cdots ,H_{QNum}$ respectively. Thereby, the adjustment is realized as the following steps:

1. Calculated the estimated parameter vector ${\hat{H}}_{CLM}$ according to the autocorrelation function vector $R_{X}(i)$;
2. Identify the corresponding subspace ${\hat{H}}_{CLM}\in H_{q},q=1,\cdots ,QNum$, and set $H_{CLM}=H_{q}$.

The above parameter quantization inevitably introduces errors into the series-indistinguishability, which affects the actual privacy-preserving performance. To ensure the effectiveness of QCLM, the following requirements should be satisfied:

1. For any subspace $H_{q},q=1,\cdots ,QNum$, it should be guaranteed that the actual privacy strength does not change significantly when replacing $H_{CLM}\in H_{q}$ with $H_{q}$;
2. It is not trivial to determine QNum. The larger the value of QNum, the smaller the quantization error, but it causes the CLM filter to change more frequently. Conversely, a smaller QNum introduces a larger quantization error, but it allows the CLM filter to remain unchanged for a longer period of time.

Obviously, it is very difficult to quantize $H_{CLM}$ without any constrains. To implement QCLM, a basic idea is to map $H_{CLM}$ to a feature parameter, and thus the parameter quantization can be achieved by dividing the definition domain of this feature parameter.

#### 4.2.2. QCLM Based on Lowpass Characteristic

Given that the autocorrelation function and the power spectrum form a Fourier pair, the power spectrum characteristics of the data series in the *X* and *Y* directions were analyzed. The results indicated that the power spectrum energy is mainly concentrated in the low frequencies (details are given in Section 5.1.4).Therefore, it is considered that series-indistinguishability can be approximated by constructing noise series with the same lowpass characteristics as the data series, and this method is called CLM-Lowpass.

In the case of the series X(I), let $S_{X}(\omega )$ denote the power spectrum at a certain moment, where ω∈[−π,π] is the normalized angle frequency, and its lowpass cutoff frequency $\omega_{X}$ can be calculated by the following equation:

(26) $$\underset{C_{ILF}\in \mathbb{R},\;\omega_{X}\in [0,\pi ]}{\arg\min}\int_{-\pi }^{\pi }{\left[C_{ILF}\cdot S_{ILF}(\omega |\omega_{X})-S_{X}(\omega )\right]}^{2}d\omega$$

where $C_{ILF}$ denotes the amplitude factor and $S_{ILF}(\omega |\omega_{X})$ denotes the power spectrum of an ideal lowpass filter with cutoff frequency at $\omega_{X}$, which is defined as follows:

(27) $$S_{ILF}(\omega |\omega_{X})=\left\{\begin{array}{lll} 1 & & \left|\omega \right|\leq \omega_{X}\\ 0 & & \omega_{X}<\left|\omega \right| \end{array}\right.$$

To approximate series-indistinguishability, the CLM filter is constructed to generate the Laplace noise series $N_{X}(I)$, whose power spectrum cutoff frequency, $\omega_{N_{X}}$, is also at $\omega_{X}$. The detailed calculation of the CLM filter’s parameters is discussed in Section 4.3.2.

**Figure 7:**
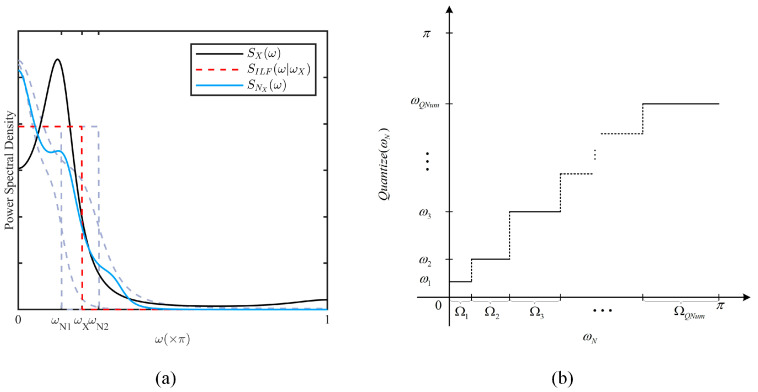
CLM-Lowpass. (**a**) Lowpass characteristics. (**b**) Quantization of noise power spectrum cutoff frequency.

As an example, the normalized power spectrum of the data and noise series, $S_{X}(\omega ),S_{N_{X}}(\omega )$, are shown in Figure 7a. Approximate series-indistinguishability does not require $S_{N_{X}}(\omega )$ to exactly match $S_{X}(\omega )$, but rather ensures that they have the same lowpass characteristics. Extensive experiments verified that, compared to original CLM, the changes in actual privacy strength induced by CLM-Lowpass are acceptable (details are given in Section 5.3.1).

In CLM-Lowpass, $H_{CLM}$ can be determined by the cutoff frequency of the noise power spectrum, $\omega_{N}$, so that the quantization of $H_{CLM}$ can be achieved by dividing $\omega_{N}$. Specifically, a basic quantization model is illustrated in Figure 7b, the normalized angular frequency interval [0, π] is divided into QNum mutually exclusive subintervals $\left\{\Omega_{1},\Omega_{2},\cdots ,\Omega_{QNum}\right\}$, which are respectively mapped to $0<\omega_{1}<\cdots <\omega_{QNum}<\pi$. It can be defined as follows:

(28) $$Quantize(\omega_{N})=\left\{\begin{array}{cc} \omega_{1} & \omega_{N}\in \Omega_{1}\\ \vdots & \vdots \\ \omega_{QNum} & \omega_{N}\in \Omega_{QNum} \end{array}\right.$$

In this way, the adaptive adjustment of the CLM filter can be implemented through the following steps:

1. Estimate the power spectrum $S_{X}(\omega )$ from the autocorrelation function vector $R_{X}(i)$ and calculate its lowpass cutoff frequency $\omega_{X}$.
2. Identify the corresponding subinterval $\omega_{X}\in \Omega_{q},q=1,\cdots ,QNum$, and set $\omega_{N}=\omega_{q}$, from which $H_{CLM}$ can be determined.

Thereby, QCLM is successfully implemented based on the lowpass characteristics of the data power spectrum, and this specific scheme is called QCLM-Lowpass. Next, we analyze the feasibility of applying QCLM-Lowpass for privacy preservation in continuous location data release.

#### 4.2.3. Feasibility Analysis of QCLM-Lowpass

As for the two-dimensional location data series L(I)=[X(I),Y(I)], the privacy mechanism M in this paper is implemented by applying QCLM-Lowpass separately in the *X* and *Y* directions. Specifically, it contains two independently operating QCLM-Lowpass components, denoted as $M_{X},M_{Y}$, and the privacy process is as follows:

1. At the release moment $t_{i}$, the Laplace noise $n_{X}(i),n_{Y}(i)\sim Lap(\lambda )$ are generated independently by $M_{X},M_{Y}$, and then the perturbed location can be obtained $\tilde{l}(i)=l(i)+[n_{X}(i),n_{Y}(i)]$.
2. During the continuous location data release, the CLM filters in $M_{X},M_{Y}$ are separately adjusted by QCLM-Lowpass, so that the autocorrelation functions of $N_{X}(I)$,X(I), and $N_{Y}(I)$, Y(I) satisfy the following condition:
  (29) $$\begin{array}{l} R_{N_{X}}(i,i-\tau )/R_{N_{X}}(i,i)=R_{XX}(i,i-\tau )/R_{XX}(i,i)\\ R_{N_{Y}}(i,i-\tau )/R_{N_{Y}}(i,i)=R_{YY}(i,i-\tau )/R_{YY}(i,i) \end{array}$$
  Here, we analyze whether M can satisfy the privacy goals presented in Section 2.3.

**a.**  **The requirement of geo-indistinguishability.**

**Theorem**  **1.** *The privacy scheme M *satisfies* $\sqrt{2}/\lambda$-geo-indistinguishability at each moment.*

**Proof of Theorem 1.**  In this scheme, the left side of Equation (12) can be expressed as follows:

$$\begin{array}{l} \frac{Pr\{M[l(i)]=\tilde{l}(i)\}}{Pr\{M[l^{\prime }(i)]=\tilde{l}(i)\}}=\frac{Lap[\tilde{x}(i)-x(i)|\lambda ]\cdot Lap[\tilde{y}(i)-y(i)|\lambda ]dxdy}{Lap[\tilde{x}(i)-x^{\prime }(i)|\lambda ]\cdot Lap[\tilde{y}(i)-y^{\prime }(i)|\lambda ]dxdy}\\ =\exp\{\frac{1}{\lambda }[\left|\tilde{x}(i)-x^{\prime }(i)\right|-\left|\tilde{x}(i)-x(i)\right|+\left|\tilde{y}(i)-y^{\prime }(i)\right|-\left|\tilde{y}(i)-y(i)\right|]\}\\ \leq \exp\{\frac{1}{\lambda }[\left|x(i)-x^{\prime }(i)\right|+\left|y(i)-y^{\prime }(i)\right|]\}\\ \leq \exp\{\frac{\sqrt{2}}{\lambda }\sqrt{{\left[x(i)-x^{\prime }(i)\right]}^{2}+{\left[y(i)-y^{\prime }(i)\right]}^{2}}\} \end{array}$$

Hence, the following result can be obtained:

$$\frac{Pr\{M[l(i)]=\tilde{l}(i)\}}{Pr\{M[l^{\prime }(i)]=\tilde{l}(i)\}}\leq \exp\{\frac{\sqrt{2}}{\lambda }d_{E}[l(i),l^{\prime }(i)]\}$$

 □

**b.**  **The requirement of series-indistinguishability.**

**Theorem** **2.** *The privacy scheme M achieves series-indistinguishability if the correlation between X(I) and Y(I) can be ignored.*

**Proof of Theorem 2.**  In this situation, the series $X(I),Y(I),N_{X}(I),N_{Y}(I)$ are independent of each other, then the following cross-correlation functions can be obtained:

$$\begin{array}{l} R_{XY}(i,i-\tau )=R_{YX}(i,i-\tau )=0\\ R_{\tilde{X}\tilde{Y}}(i,i-\tau )=R_{\tilde{Y}\tilde{X}}(i,i-\tau )=0 \end{array}$$

According to Equation (29), the normalized autocorrelation functions of $\tilde{X}(I),\tilde{Y}(I)$ satisfy the following:

$$\begin{array}{l} \frac{R_{\tilde{X}\tilde{X}}(i,i-\tau )}{R_{\tilde{X}\tilde{X}}(i,i)}=\frac{R_{XX}(i,i-\tau )+R_{N_{X}}(i,i-\tau )}{R_{XX}(i,i)+R_{N_{X}}(i,i)}=\frac{R_{XX}(i,i-\tau )}{R_{XX}(i,i)}\\ \frac{R_{\tilde{Y}\tilde{Y}}(i,i-\tau )}{R_{\tilde{Y}\tilde{Y}}(i,i)}=\frac{R_{YY}(i,i-\tau )+R_{N_{Y}}(i,i-\tau )}{R_{YY}(i,i)+R_{N_{Y}}(i,i)}=\frac{R_{YY}(i,i-\tau )}{R_{YY}(i,i)} \end{array}$$

Thus, the autocorrelation function matrix of $\tilde{L}(I)$ satisfies the following:

$$\begin{array}{ll} R_{\tilde{L}}(i,i-\tau )⊘R_{\tilde{L}}(i,i) & =\left[\begin{array}{cc} R_{\tilde{X}\tilde{X}}(i,i-\tau )/R_{\tilde{X}\tilde{X}}(i,i) & 0\\ 0 & R_{\tilde{Y}\tilde{Y}}(i,i-\tau )/R_{\tilde{Y}\tilde{Y}}(i,i) \end{array}\right]\\ & =\left[\begin{array}{cc} R_{XX}(i,i-\tau )/R_{XX}(i,i) & 0\\ 0 & R_{YY}(i,i-\tau )/R_{YY}(i,i) \end{array}\right]\\ & =R_{L}(i,i-\tau )⊘R_{L}(i,i) \end{array}$$

Thus, $L(I),\tilde{L}(I)$ are series-indistinguishable. □

**c.**  **The analysis of quantization errors.**

This paper considers the more robust series-indistinguishability defined by Equation (7) and analyzes the effect of quantization errors introduced by QCLM-Lowpass on series-indistinguishability.

In some works [20,21], the location data series was described using a low-order autoregressive model. Consequently, this paper assumed that the noise series N satisfies a second order autoregressive model, and its system function can be expressed as follows:

(30) $$H_{N}(z)=\frac{1}{\left(1-\xi_{1}z^{-1})(1-\xi_{2}z^{-1}\right)}$$

where $\xi_{1},\xi_{2}$ are the poles. It is considered that the cutoff frequency of the noise power spectrum $\omega_{N}\in (0.05\pi ,0.8\pi )$, and $\xi_{1},\xi_{2}$ can be calculated by the following function:

(31) $$\underset{\left|\xi_{1}\right|,\left|\xi_{2}\right|\leq 1,\;C_{N}\in \mathbb{R}}{\arg\min}\int_{-\pi }^{\pi }{\left[C_{N}\cdot {\left\|H_{N}(z){|}_{z=e^{j\omega }}\right\|}_{2}^{2}-S_{ILF}(\omega |\omega_{N})\right]}^{2}d\omega$$

where $C_{N}$ denotes the amplitude coefficient. It is found that the results of Equation (31) are conjugate complex poles with the modulus and azimuth, γ,θ, which can be approximated in terms of $\omega_{N}$:

(32) $$\begin{array}{l} \gamma =\sqrt{\xi_{1}\xi_{2}}\approx 0.0487{\omega_{N}}^{2}-0.3544\omega_{N}+0.9802\\ \theta =\arccos(\frac{\xi_{1}+\xi_{2}}{2\sqrt{\xi_{1}\xi_{2}}})\approx -0.1058{\omega_{N}}^{2}+0.7907\omega_{N}-0.0222 \end{array}$$

**Figure 8:**
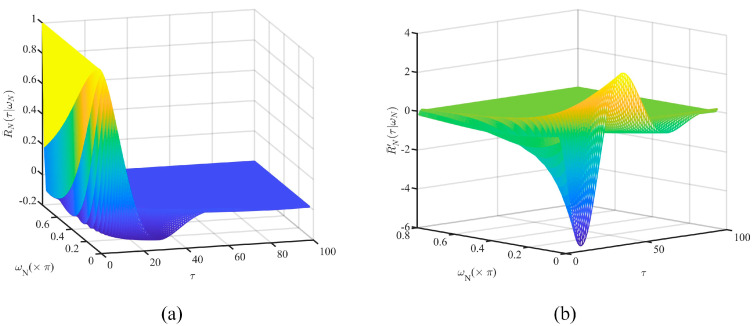
The results of the autocorrelation function in AR model. (**a**) ${\bar{R}}_{N}(\tau |\omega_{N})$. (**b**) ${\bar{R}}_{N}^{\prime }(\tau |\omega_{N})$.

In this case, the normalized autocorrelation function, ${\bar{R}}_{N}(\tau |\omega_{N})$, can be expressed as follows:

(33) $${\bar{R}}_{N}(\tau |\omega_{N})=\gamma^{\tau }[\frac{1-\gamma^{2}}{1+\gamma^{2}}\cot(\theta )\sin(\tau \theta )+\cos(\tau \theta )]$$

where τ∈ℕ denotes the lag of the autocorrelation function. As shown in Figure 8a, the larger the value of $\omega_{N}$, the faster ${\bar{R}}_{N}(\tau |\omega_{N})$ decays to 0.

Hence, the difference in autocorrelation function caused by the quantization error Δω can be expressed as follows:

(34) $$\Delta {\bar{R}}_{N}(\tau |\omega_{N},\Delta \omega )={\bar{R}}_{N}(\tau |\omega_{N}+\Delta \omega )-{\bar{R}}_{N}(\tau |\omega_{N})={\bar{R}}_{N}^{\prime }(\tau |{\tilde{\omega }}_{N})\cdot \Delta \omega$$

where the mean value theorem is utilized, $\left|{\tilde{\omega }}_{N}-\omega_{N}\right|\leq \left|\Delta \omega \right|$, and ${\bar{R}}_{N}^{\prime }(\tau |\omega_{N})$ is defined as follows:

(35) $$\begin{array}{r} {\bar{R}}_{N}^{\prime }(\tau |\omega_{N})=\frac{d{\bar{R}}_{N}(\tau |\omega_{N})}{d\omega_{N}}=\gamma^{\tau -1}[\tau \cos(\tau \theta )-\frac{\tau \gamma^{4}+4\gamma^{2}-\tau }{{\left(1+\gamma^{2}\right)}^{2}}\cot(\theta )\sin(\tau \theta )]\frac{d\gamma }{d\omega_{N}}\\ +\gamma^{\tau }[\frac{1-\gamma^{2}}{1+\gamma^{2}}\frac{\tau \sin(2\theta )\cos(\tau \theta )-2\sin(\tau \theta )}{2\sin^{2}(\theta )}-\tau \sin(\tau \theta )]\frac{d\theta }{d\omega_{N}} \end{array}$$

By substituting Equation (32) into Equation (35), the approximation of ${\bar{R}}_{N}^{\prime }(\tau |\omega_{N})$ can be obtained as shown in Figure 8b. Thereby, the following conclusions can be drawn:

1. $\Delta {\bar{R}}_{N}(\tau |\omega_{N},\Delta \omega )$ is bounded with respect to Δω.
2. $\Delta {\bar{R}}_{N}(\tau |\omega_{N},\Delta \omega )$ is not linearly related to Δω and shows a tendency to decay to 0 as $\omega_{N}$, τ increase.

Based on the same analytical approach, the above conclusions can be generalized to higher-order models.

In summary, the impact of the quantization error in QCLM-Lowpass on series-indistinguishability is controllable. Based on this quantitative relationship, the deviation of series-indistinguishability can be kept within the expected range by setting the quantization scheme. In addition, the division of $\omega_{N}$ should not be uniform. Intuitively, the granularity of the division is finer in the low-frequency part, while the high-frequency part can be relaxed.

### 4.3. Algorithmic Implementation

There are some problems in the application of QCLM-Lowpass that should be discussed, and then the algorithmic implementation is given.

#### 4.3.1. Quasi-Stationary State Identification

The correlation estimation method proposed in Section 4.1 is applicable only if the location increments satisfy the stationary conditions. Therefore, it is necessary to identify the stationary state of the location increments.

In this paper, the stationary conditions are relaxed to allow the mean and autocorrelation function to vary within a reasonable range, called the quasi-stationary state, denoted as QS. Considering that V(I) is stationary when the user is moving in uniform linear motion, the quasi-stationary state is identified based on the change of the location increments’ modulus $\left|v(i)\right|$ and azimuth φ(i):

(36) $$\left\{\begin{array}{c} \left|v(i)\right|=\sqrt{{\left[v_{X}(i)\right]}^{2}+{\left[v_{Y}(i)\right]}^{2}}\\ \varphi (i)=\arctan[v_{Y}(i)/v_{X}(i)] \end{array}\right.$$

For the data in window with size $M_{V}+1$, $V_{W}(i)={\left[v(i-M_{V}),\cdots ,v(i)\right]}^{T}$, let ${\left|v\right|}_{i}^{\prime },\;{\left|v\right|}_{i}^{\prime \prime }$ denote the minimum and maximum modulus and $\Delta \varphi_{i}$ denote the maximum change in azimuth:

(37) $${\left|v\right|}_{i}^{\prime }=\underset{k\in [0,M_{V}]}{\min}[|v(i-k)|]$$

(38) $${\left|v\right|}_{i}^{\prime \prime }=\underset{k\in [0,M_{V}]}{\max}[|v(i-k)|]$$

(39) $$\Delta \varphi_{i}=\underset{k,m\in [0,M_{V}]}{\max}angle[\varphi (i-k)-\varphi (i-m)]$$

where $angle(\theta_{k},\theta_{m})$ is the angle between two azimuths:

(40) $$angle(\theta_{k}-\theta_{m})=\left\{\begin{array}{cc} \left|\theta_{k}-\theta_{m}\right| & \left|\theta_{k}-\theta_{m}\right|\leq \pi \\ 2\pi -\left|\theta_{k}-\theta_{m}\right| & other \end{array}\right.$$

In the quasi-stationary state, ${\left|v\right|}_{i}^{\prime },\;{\left|v\right|}_{i}^{\prime \prime },\;\Delta \varphi_{i}$ should satisfy the following conditions:

(41) $$\Delta \varphi_{i}\leq \Phi_{QS}$$

(42) $${\left|v\right|}_{i}^{\prime \prime }-{\left|v\right|}_{i}^{\prime }\leq \eta_{QS}^{\prime }\cdot \sum_{m=1}^{M_{V}}\left|v(i-m)\right|$$

(43) $${\left({\left|v\right|}_{i}^{\prime \prime }\right)}^{2}-{\left({\left|v\right|}_{i}^{\prime }\right)}^{2}\leq \eta_{QS}^{\prime \prime }\cdot \sum_{m=1}^{M_{V}}{\left[|v(i-m)|\right]}^{2}$$

where $\Phi_{QS},\eta_{QS}^{\prime },\eta_{QS}^{\prime \prime }$ are the thresholds for relative changes in azimuth, modulus, and squared modulus, respectively. Intuitively, the smaller the value of $\Phi_{QS},\eta_{QS}^{\prime },\eta_{QS}^{\prime \prime }$, the more stationary the series V(I).

To reduce the effects of estimation errors or transient changes in the data, the actual state is determined based on the estimated results over a period of time. Let $\hat{S}(i)$ denote the estimated state from Equations (41)–(43) at the moment $t_{i}$, and S(i) denote the actual state. As for the estimated results in the window with the size of $M_{\hat{S}}$, if any of the following conditions hold:

(44) $$\forall k\in [0,M_{\hat{S}}-1]:\hat{S}(i-k)=QS$$

(45) $$S(i-1)=QS\;and\;\exists k\in [0,M_{\hat{S}}-1]:\hat{S}(i-k)=QS$$

then the actual state S(i)=QS.

In addition, there are inevitable errors in V(I), which affect the accuracy of the quasi-stationary state identification and the location correlation estimation. Therefore, the noise reduction is necessary to estimate the true location increments. However, how to achieve the adaptive filtering for V(I) is not the focus of this paper and is not discussed in detail here.

#### 4.3.2. CLM Filter Design

The CLM filter is constructed by an all-pole filter, and the system function is as follows:

(46) $$H_{CLM}(z)=\frac{1}{\sum_{m=0}^{O_{CLM}}a_{m}z^{-m}}=\frac{1}{\prod_{m=0}^{O_{CLM}}\left(1-\xi_{m}z^{-1}\right)}$$

where $O_{CLM}$ denotes the filter order, the parameter vector $A_{CLM}=[a_{1},\cdots ,a_{O_{CLM}}]$ while $a_{0}=1$. Let $\Xi_{CLM}={\left[\xi_{1},\cdots ,\xi_{O_{CLM}}\right]}^{T}$ denote the pole vector. To ensure the stability of the filter, it should be satisfied that $|\xi_{m}|<1,\;m=1,\cdots ,O_{CLM}$.

According to Equation (9) with the convolution theorem, it can be seen that the Laplace noise series’ power spectrum $S_{N}(\omega )$ is the result of the convolution of the correlated Gaussian noise’ power spectrum $S_{G^{\prime }}(\omega )$ with itself. In the interval [0,2π], it behaves as a circular convolution, which can be expressed as follows:

(47) $$S_{N}(\omega )=\frac{4}{\pi }\int_{0}^{2\pi }S_{G^{\prime }}(\upsilon )\cdot S_{G^{\prime }}[{\left(\omega -\upsilon \right)}_{mod\;2\pi }]d\upsilon$$

and $S_{G^{\prime }}(\omega )$ can be calculated as follows:

(48) $$S_{G\prime }(\omega )=\sigma_{G}^{2}\cdot {\left\|H_{CLM}(z){|}_{z=e^{j\omega }}\right\|}_{2}^{2}$$

Similar to Equation (26), the poles vector $\Xi_{CLM}$ can be computed by allowing $S_{N}(\omega )$ to approximate $S_{ILF}(\omega |\omega_{N})$, which can be expressed as follows:

(49) $$\underset{C_{N}\in \mathbb{R},\;\Xi_{CLM}}{\arg\min}\int_{-\pi }^{\pi }{\left[C_{N}\cdot S_{N}(\omega |\Xi_{CLM})-S_{ILF}(\omega |\omega_{N})\right]}^{2}d\omega$$

where $C_{N}$ denotes the amplitude coefficient, and the CLM filter is limited to a lowpass filter. In this way, the parameter vector $A_{CLM}$ can be determined by the cutoff frequency $\omega_{N}$.

In QCLM-Lowpass, the CLM filter is set with different levels by parameter quantization, and these levels, corresponding to $\omega_{N}=\omega_{1},\omega_{2},\cdots ,\omega_{QNum}$, are respectively denoted as L=1,2,⋯,QNum. Intuitively, the correlation of the Laplace noise series will decrease as L increases. In practice, a matrix with size $QNum\times O_{CLM}$, denoted as Param, is used to store the parameters of the CLM filter in different levels, where each row corresponds to one level. The level L can be used as the index to obtain corresponding parameter vector $A_{CLM}=Param(L)$.

#### 4.3.3. Quantization Scheme

Recalling the basic requirements presented in Section 4.2.1 and the conclusions on quantization error in Section 4.2.3, based on experiments with real data, it is empirically found that the following quantization scheme achieves a better privacy-preserving effect,

(50) $$Quantize(\omega_{N})=\left\{\begin{array}{cc} 0.1\pi & \omega_{N}\in \left(0,0.1\pi \right]\\ 0.125\pi & \omega_{N}\in \left(0.1\pi ,0.15\pi \right]\\ 0.175\pi & \omega_{N}\in \left(0.15\pi ,0.2\pi \right]\\ 0.25\pi & \omega_{N}\in \left(0.2\pi ,0.3\pi \right]\\ 0.35\pi & \omega_{N}\in \left(0.3\pi ,0.4\pi \right]\\ 0.45\pi & \omega_{N}\in \left(0.4\pi ,\pi \right] \end{array}\right.$$

Note that the value of $Quantize(\omega_{N})$ is limited between 0.1π and 0.45π. This is because when $\omega_{N}$ is less than 0.1π, the poles of the CLM filter are close to the unit circle, and it takes longer for the filter to return to the steady state after adjustment. This affects the real-time processing performance of the scheme and also tends to make the filter unstable. In addition, due to the limited filter order, there is a trailing phenomenon in the noise power spectrum. As $\omega_{N}$ increases, there will be some high-frequency noise that can be easily filtered out. Therefore, considering the power spectrum characteristics of real data, $\omega_{N}$ is limited to no more than 0.45π.

#### 4.3.4. Level Identification

To satisfy the demands of real-time processing, it is necessary for the privacy mechanism to quickly determine the level L of the CLM filter. Given that $R_{N}(\tau )$ and $S_{N}(\omega )$ are Fourier transform pairs, there is a certain correspondence between them in the shape of the curves. Intuitively, the steeper the curve of $R_{N}(\tau )$, the flatter the curve of $S_{N}(\omega )$. Therefore, a linear regression is performed on $R_{N}(\tau )$ and the opposite of its slope, denoted as χ, is considered as the feature parameter for level identification:

(51) $$\chi =\frac{6ϒ\sum_{\tau =0}^{ϒ}R_{N}(\tau )-12\sum_{\tau =0}^{ϒ}\tau \cdot R_{N}(\tau )}{ϒ(ϒ+1)\left(ϒ+2\right)R_{N}(0)}$$

where ϒ denotes the maximum lag of $R_{N}(\tau )$. The larger the value of χ, the steeper the curve of $R_{N}(\tau )$ and the lower the correlation of the noise series, requiring a higher level of the CLM filter.

Based on extensive real data, the distribution of χ corresponding to different quantized intervals in Equation (50) is analyzed, where ϒ=3. The details are given in Section 5.3.3. The following level identification method L=Identify(χ) is empirically obtained:

(52) $$Idenfity(\chi )=\left\{\begin{array}{cc} 1 & \chi \in [0,0.1055)\\ 2 & \chi \in [0.106,0.1855)\\ 3 & \chi \in [0.1855,0.2235)\\ 4 & \chi \in [0.2235,0.3595)\\ 5 & \chi \in [0.3595,0.4705)\\ 6 & \chi \in [0.4705,1) \end{array}\right.$$

Similar to the quasi-stationary state identification, the actual level of the CLM filter is determined based on the estimated results over a period of time. Let $\hat{L}(i)$ denote the estimated level from Equation (52) at the moment $t_{i}$, and the estimated level change is denoted as $\psi (i)=\mathrm{sgn}[\hat{L}(i)-\hat{L}(i-1)]$, where sgn(⋅) is sign function. Then, the results in the window with the size of $M_{\Psi }+1$ are used to determine the actual level L(i). Specifically, the CLM filter is adjusted only if $\forall m\in [0,M_{\Psi }-1]:\psi (i-m)=0$:

(53) $$L(i)=L(i-1)+\psi (i-M_{\Psi })$$

In this way, the CLM filter can only be adjusted between adjacent levels to avoid drastic changes.

#### 4.3.5. Gain Coefficients

As described in Section 3.2.2, due to the transient response, the actual output cannot be normalized based on the steady-state response during the transition phase. Therefore, it is necessary to set the gain coefficient vector $K={\left[\kappa_{0},\cdots ,\kappa_{M_{T}}\right]}^{T}$ for the transition phase, where $M_{T}$ is the window size of the transition phase, while $\kappa_{m},m=0,\cdots ,M_{T}$ denotes the gain coefficient at the m-th moment after adjustment; in particular, let $\kappa_{M_{T}}=\kappa_{CLM}$ given in Equation (11).

Due to the parameter quantization, K can be easily obtained. More specifically, by simulating the CLM filter switched between different levels, the Laplace scales of the actual output at different moments can be estimated, so that the reasonable value of $M_{T}$ and K can be determined.

A matrix with size $QNum\times QNum\times (M_{T}+1)$, denoted as GainCoeff, is used to store the gain coefficients in different level shifts, and $\left(L_{1},L_{2}\right)$ is used to as the index to obtain the gain coefficient vector, $K=GainCeoff(L_{1},L_{2})$, which corresponds to the CLM filter switching from level $L_{1}$ to level $L_{2}$.

#### 4.3.6. Implementation of QCLM-Lowpass

Similar to DCLM, QCLM-Lowpass is considered to be a program object in which CLM is a member. The main attributes and methods are given in Table 3.

At the beginning of the application, the method Initialization is used to set the attributes of QCLM-Lowpass and initialize CLM. By default, the CLM filter’s initial level is set as QNum, and its parameter vector and gain coefficient are set accordingly. At each moment, the method Iteration is called to adjust the CLM filter and generate the Laplace noise, and its technical description is given in Algorithm 3.

**Algorithm 3.** QCLM-Lowpass: Iteration**Input**: the autocorrelation function vector R**Output**: the Laplace noise n1Calculate the feature parameter χ according to R;2Calculate the estimated level ${\hat{L}}_{CLM}$ according to χ,

${\hat{L}}_{CLM}=Identify(\chi )$

3Update Ψ,

$\begin{array}{cc} \psi_{m-1}\leftarrow \psi_{m} & m=2,\cdots ,M_{\Psi }+1\\ \psi_{m}\leftarrow \mathrm{sgn}({\hat{L}}_{CLM}-\hat{L}) & m=M_{\Psi }+1 \end{array}$

4**if** $\iota \leq M_{T}$ **then**5  Keep the actual level unchanged $L_{CLM}=L$;6  Update the index ι=ι+1;7  Set the gain coefficient κ=K(ι);8  Update the gain coefficient of CLM

CLM.UpdateGainCoeff(κ)

9**elseif** $\psi_{1}\neq 0$ and $\sum_{m=2}^{M_{\Psi }+1}\left|\psi_{m}\right|=0$ **then**10  Calculate the actual level $L_{CLM}=L+\psi_{1}$;11  Calculate the parameter vector $A=Param(L_{CLM})$;12  Calculate the gain coefficient vector $K=GainCoeff(L,L_{CLM})$;13  Set ι=1, and the gain coefficient κ=K(ι)14  Update the parameters of CLM

$\begin{array}{l} CLM.UpdateParam([],A)\\ CLM.UpdateGainCoeff(\kappa ) \end{array}$

15
**else**
16  Keep the actual level unchanged $L_{CLM}=L$;17
**end if**
18Update $\hat{L},L$,

$\begin{array}{l} \hat{L}\leftarrow {\hat{L}}_{CLM}\\ L\leftarrow L_{CLM} \end{array}$

19Generate the Laplace noise n=CLM.LaplaceGeneration( )20**return** *n*

Lines 1–3 calculate the feature parameter χ, from which the estimated level ${\hat{L}}_{CLM}$ is obtained, and then update the estimated level change record. In lines 4–8, when the CLM filter is in the transient phase, only the gain coefficient is updated without adjusting the parameter vector. When the CLM filter is in the steady state, the estimated level change record is used to determine whether a parameter adjustment is required, and the conditions are shown in line 9. If it is, then the actual level of the CLM filter is calculated, and its parameters and the gain coefficient are updated in lines 10–14; otherwise, the filter is left unchanged in line 15. Finally, CLM is called to generate the Laplace noise and return in lines 19–20.

## 5. Experimental Evaluation

In this study, we conducted experiments on real-life datasets to evaluate QCLM-Lowpass. First, the stationarity and the power spectrum characteristics of the actual location data were analyzed. Then, the effectiveness of QCLM-Lowpass was verified. Finally, its performance in privacy protection and data availability were evaluated. All experiments were performed using MATLAB 2023a on a computer with the Intel(R) Xeon(R) CPU E3-1240 v5 @ 3.50GHz and 16GB of memory.

### 5.1. Experimental Datasets

#### 5.1.1. Real Datasets

The following three real-life datasets were used in experiments:

1. GeoLife [22,23,24]. The dataset contains trajectory data from 182 volunteers between April 2007 and August 2012, containing 17,621 trajectories with a total distance span of 1,292,951 km.
2. T-Drive [25,26]. This dataset collects the trajectories of 10,357 taxis in Beijing between 2 February 2008 and 8 February 2008, and contains more than $1.5\times 10^{7}$ location points with a total distance of $9\times 10^{6}$ km.
3. OpenStreetMap(OSM) (https://www.openstreetmap.org/traces, accessed on 17 July 2022). It is a collaborative online mapping project that allows users to share their trajectories. We downloaded 406,399 trajectories with more than $1.7\times 10^{9}$ locations between May 2016 and May 2022, including location data with high-frequency sampling (less than 1 s) and high accuracy (less than 1 m).

#### 5.1.2. Data Preprocessing

Since increased time interval Δt leads to decreased data correlation and the appearance of spectral aliasing, this paper required that 1≤Δt≤10. The location series satisfying this condition were extracted from real datasets with linear interpolation, which was constrained by two conditions: (1) consecutive interpolations cannot exceed two times; (2) total interpolations cannot exceed 20% of the current data series length. After applying a length threshold of 200, a total of 177,089 location series were retained for analysis.

**Figure 9:**
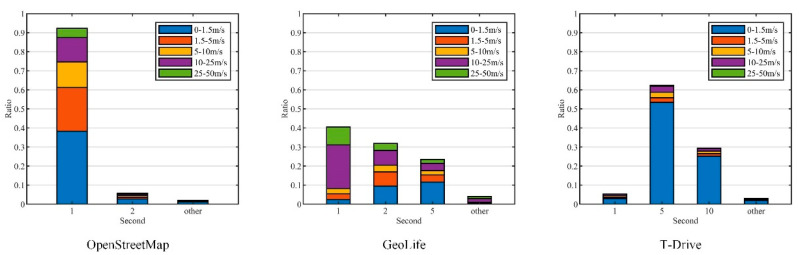
The distribution of sampling intervals and velocity.

Figure 9 presents the distribution of sampling intervals and velocity in different datasets. In OpenStreetMap, approximately 92.3% of the data is collected with a sampling interval of 1 s, with 38.25% of these observations corresponding to low-velocity conditions (0–1.5 m/s). In the GeoLife dataset, the sampling intervals predominantly occurred at 1 s (40.55%), 2 s (31.89%), and 5 s (23.51%), covering a range of motion scenarios with varying velocities. Conversely, the T-Drive dataset exhibited sampling intervals chiefly at 5 s (62.30%) and 10 s (29.44%), which are typically associated with low-velocity conditions. These results demonstrate the diversity of the experimental data and reflect the applicability of the experimental conclusions in this paper.

#### 5.1.3. Stationary Analysis

Recall from Section 4.3.1 that the location series whose increments satisfy the quasi-stationary state conditions were screened out, where the quasi-stationary state thresholds were set as $\Phi_{QS}=5\pi /36,\;\eta_{QS}^{\prime }=\eta_{QS}^{\prime \prime }=0.1$ and the window size for estimation and state identification were $M_{V}=\left\lfloor 60/\Delta t\right\rfloor$ and $M_{\hat{S}}=\left\lfloor 30/\Delta t\right\rfloor$.

**Figure 10:**
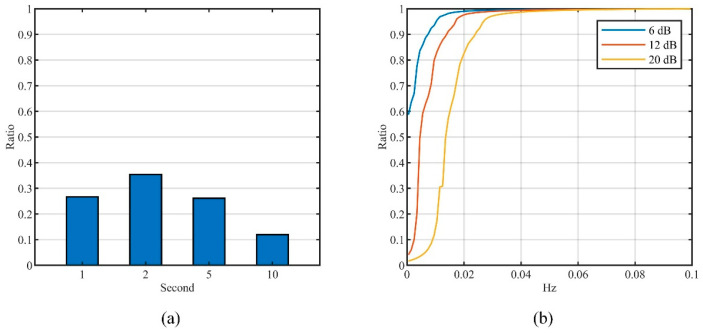
Statistic results. (**a**) Distribution of the data series with quasi-stationary increments. (**b**) Cumulative distribution of attenuation frequencies.

Figure 10a presents the results in different sampling intervals. In the real datasets covered in this paper, the data series with quasi-stationary increments occupy 27.46%, where the results in the sampling intervals of 1 s, 2 s, and 5 s are higher than 25%. This indicates that the correlation estimation method proposed in this paper has a wider range of applicable scenarios.

Afterwards, segments with lengths less than 200 were filtered out, resulting in 134,691 location series for subsequent experiments.

#### 5.1.4. Power Spectrum Characterization

Based on the correlation estimation method in Section 4.1, the power spectrum characteristics of real location data were analyzed using a third order AR model, where the estimation window size was set as $M_{L}=\left\lfloor 60/\Delta t\right\rfloor$. The power spectrum of each sliding window in the *X* and *Y* directions was estimated and its 6 dB, 12 dB, and 20 dB attenuation frequency were counted, and the cumulative distribution are shown in Figure 10b. Results reveals that the energy of the power spectrum is mainly concentrated in the low frequency part, where the 20 dB attenuation frequency is mainly distributed below 0.04 Hz. To achieve series-indistinguishability, the noise power spectrum should also satisfy this lowpass characteristics, which is the basis of QCLM-Lowpass.

### 5.2. Experimental Configurations

#### 5.2.1. Competitors

To evaluate the performance of QCLM-Lowpass, we compare it with several CLM filter adjustment schemes and a representative privacy scheme based on the Markov model:

1. IID. This is the classical Laplace mechanism that adds independently and identically distributed Laplace noise to the data series, which is used as the basic reference in this paper.
2. DCLM. Recall from Section 3.1 that this scheme calculates the CLM filter parameter directly based on the estimated data correlation, and thus dynamically adjusts the filter. In the experiments, the adjustment step is set as $u_{DCLM}=0.01$.
3. NonQCLM. In contrast to QCLM-Lowpass, this scheme dynamically adjusts the noise power spectrum cutoff frequency $\omega_{N}$ instead of quantizing it, with the adjustment step being set as $\mu_{\omega }=0.01$ in experiments.
4. Markov-GRR, which was proposed in [17]. The setup is described in Section 5.4.

In DCLM, NonQCLM, and QCLM-Lowpass, the CLM filter was implemented using a third order all-pole filter, with initial parameter values set according to the highest level in QCLM-Lowpass. The window size of correlation estimation was set as $M_{L}=\left\lfloor 60/\Delta t\right\rfloor$. The window size of level identification and transition phase in QCLM-Lowpass were set as $M_{\Psi }=\left\lfloor 30/\Delta t\right\rfloor$ and $M_{T}=30$ respectively.

#### 5.2.2. Evaluation Metrics

The following metrics were used to evaluate the effectiveness of the privacy scheme with respect to data availability and privacy protection.

The data availability was measured by the mean perturbation distance (MPD):

(54) $$MPD=\frac{1}{\left|L\right|}\sum_{l\in L}d_{E}(l,\tilde{l})$$

where |L| denotes the num of locations, $l,\tilde{l}$ denote the original and perturbed location, respectively. This metric is independent of the specific application and can therefore be considered a generalized availability evaluation metric [16]. Intuitively, the larger the MPD value, the more the data is disturbed, resulting in a greater loss of data availability.

To measure the privacy protection strength for the single location l, we calculated the $\varepsilon_{l}$-geo-indistinguishability achieved by the privacy scheme within the region $Area=\{l^{\prime }|d_{E}(l^{\prime },l)\leq r_{eff}\}$, i.e., for $\forall \tilde{l},{\tilde{l}}^{\prime }\in Area$, the privacy scheme M satisfies the following inequation:

(55) $$\frac{Pr[M(l)=\tilde{l}]}{Pr[M(l)={\tilde{l}}^{\prime }]}\leq \exp[\varepsilon_{l}\cdot d_{E}(\tilde{l},{\tilde{l}}^{\prime })]$$

where $r_{eff}$ denotes the radius of the focus area.

In addition, for the location dataset, $L_{Set}$, we characterized its overall privacy strength, $E_{\phi },0\leq \phi \leq 1$, using the privacy strength satisfied by most locations in it, that is, for $\forall l\in L_{Set}$:

(56) $$Pr(\varepsilon_{l}\leq E_{\phi })\geq 1-\phi$$

Specifically, $E_{0}$ indicates the worst-case privacy loss. In this way, the impact of individual statistical results on the overall evaluation results can be reduced.

In the experiment, each privacy mechanism was repeated 500,000 times and the distribution of the noise at each moment was counted, from which MPD and the privacy strength was calculated.

#### 5.2.3. Filtering Attack

The filtering attack [12] was used to evaluate the actual effectiveness of the privacy scheme. The difference in privacy strength before and after the attack indicates the ability to resist the correlation-based attack.

Specifically, the 20 dB attenuation frequency of the data power spectrum is considered the cutoff frequency, and a fourth order Butterworth model is adopted to design the lowpass filter to implement the attack, where the minimum normalized cutoff frequency is set as 0.1π. For the time-varying data correlation, it is necessary to adjust the lowpass cutoff frequency accordingly, and the adjustment step was set to 0.05 in the experiment.

### 5.3. Validity Analysis of QCLM-Lowpass

There are two simplified operations in QCLM-Lowpass: CLM-Lowpass and parameter quantization. First, the effectiveness of CLM-Lowpass was analyzed, and then the effect of parameter quantization on privacy strength was analyzed. Finally, the performance of QCLM-Lowpass was demonstrated in continuous location data release.

#### 5.3.1. Effectiveness of CLM-Lowpass

To validate the CLM-Lowpass, extensive stationary one-dimensional data series with different power spectra were simulated based on the results in Section 5.1.4, and then the privacy strength of original CLM and CLM-Lowpass were compared under the filtering attack.

**Figure 11:**
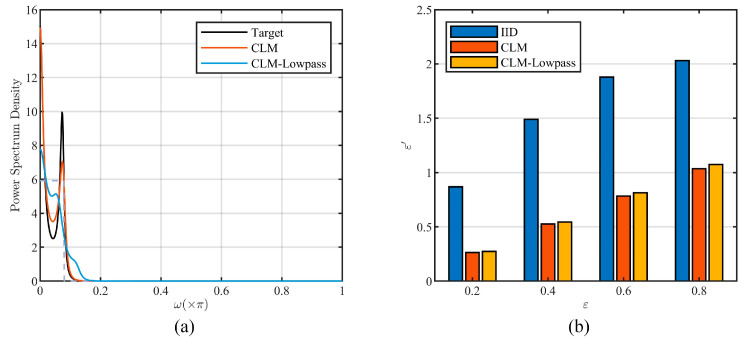
Comparison results between the original CLM and CLM-Lowpass. (**a**) The noise power spectrum. (**b**) The actual privacy strength under the filtering attack.

Figure 11 illustrates the results of one simulation experiment, with the power spectrum of the target data and noise series shown in Figure 11a, where the lowpass characteristic is marked by the dashed line.

In this experiment, the length of simulated data series was 1000 and the privacy budget and sensitivity were set as ε = 0.2, 0.4, 0.6, and 0.8 and Δf=10, while δ=0.05 in the calculation of differential privacy strength $\varepsilon^{\prime }$ under filtering attack. Figure 11b presents the results of different privacy schemes. The results of original CLM and CLM-Lowpass are very close, and the maximum relative percentage difference between them is 4.03%. This indicates that there is no significant difference between the two methods with the significance criterion of 5%. And this conclusion was supported by all simulation experiments. Therefore, CLM-Lowpass is feasible in the scenarios covered in this paper.

#### 5.3.2. Effectiveness of Parameter Quantization

To analyze the effect of parameter quantization on the privacy strength, we compared the privacy strength achieved by noise series with different lowpass cutoff frequencies under the filtering attack.

In the experiment, the lowpass cutoff frequency of the data power spectrum, $\omega_{X}$, was simulated to vary from 0.05π to 0.5π with the interval 0.05π. The corresponding lowpass filters were constructed for the filtering attack, and the noise series with the same cutoff frequency were generated by CLM-Lowpass while IID was considered as a reference, resulting in 110 different combinations. The length of the simulated data series was 1000, the scale of Laplace distribution and sensitivity were λ=20 and Δf=10, and δ=0.05 in the calculation of differential privacy strength $\varepsilon^{\prime }$ under filtering attack.

Here, the combination where the noise series has the same cutoff frequency as the data series is referred to as the series-indistinguishable scheme, denoted as SI. Under the same filtering attack, we computed the absolute value of the relative percentage difference, |RPD|, between different combinations with SI in terms of privacy strength $\varepsilon^{\prime }$, which can be calculated as follows:

(57) $$|RPD|=\frac{\left|\varepsilon^{\prime }-\varepsilon_{SI}^{\prime }\right|}{\varepsilon_{SI}^{\prime }}\times 100$$

where $\varepsilon_{SI}^{\prime }$ denotes the privacy strength in SI after the filtering attack.

**Figure 12:**
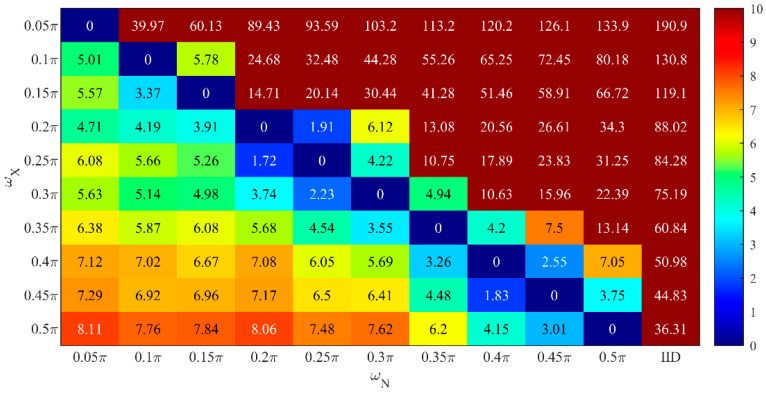
|*RPD*| (%).

As shown in Figure 12, only the results in the same row can be compared, where |RPD|=0 corresponds to SI under the current filtering attack. There are some combinations close to SI that have absolute percentage differences of less than 5%, which are acceptable in practical applications. This indicates that the actual privacy strength can be acceptably maintained despite variations in the noise power spectrum induced by the parameter quantization. These results also provide a basis for the parameter quantization scheme given in Equation (50).

#### 5.3.3. Feature Parameter in Level Identification

Recall from Section 4.3.4 that the feature parameter χ defined in Equation (51) is used to identify the CLM filter’s level in QCLM-Lowpass. Based on the real dataset, the distribution of χ corresponding to different levels was analyzed. The autocorrelation function and the power spectrum are estimated as in Section 5.1.4, while the max lag of the autocorrelation function was set as ϒ=3.

**Figure 13:**
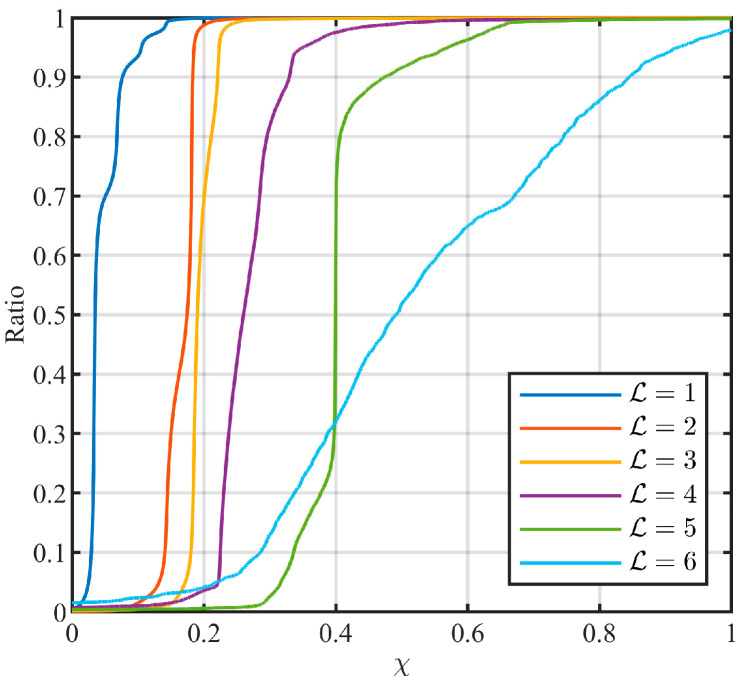
Cumulative distribution of the feature parameter χ.

As can be seen from Figure 13, there are obvious differences between the cumulative distributions of χ corresponding to different levels. This indicates that χ is effective for level identification. Taking the ratio of 0.95 as a criterion, the level identification method given in Equation (52) was determined.

#### 5.3.4. The Effectiveness of QCLM-Lowpass for Continuous Location Data Publishing

**Figure 14:**
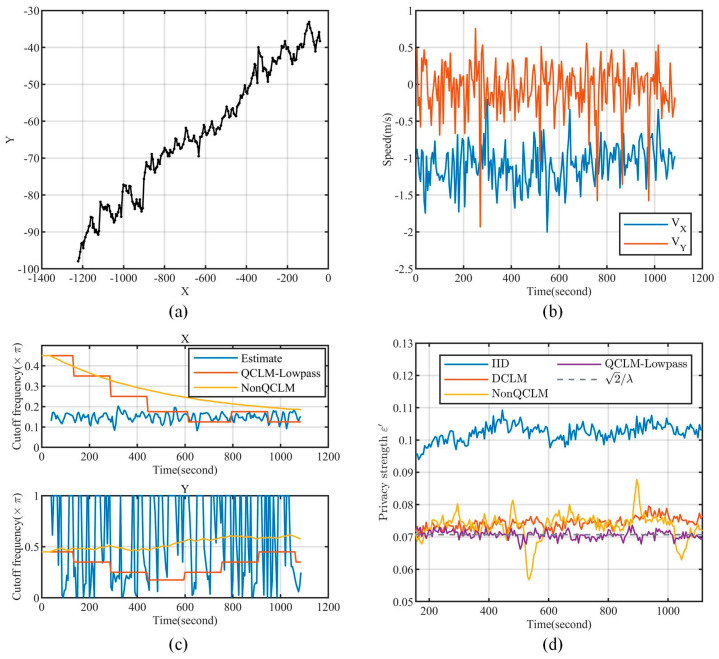
Comparison results in continuous location release. (**a**) Location series. (**b**) Velocity on the X, Y directions. (**c**) The cutoff frequency of noise power spectrum over time. (**d**) The privacy strength at each moment after the filtering attack.

To demonstrate the performance of QCLM-Lowpass, we simulated a continuous publishing scenario using a randomly selected location series with a sampling interval of 5 s. Figure 14a,b show the location series and the velocity–time curve in the *X* and *Y* directions, respectively. Figure 14c demonstrates the cutoff frequency of the noise power spectrum in the QCLM-Lowpass and NonQCLM in the *X* and *Y* directions. It can be seen that both methods can track the changes of the data power spectrum, but QCLM-Lowpass based on parameter quantization allows the CLM filter to remain in the steady state for a longer time.

Under the setting of the Laplace distribution scale λ=20, radius of protected area $r_{eff}=50$ m, we compared the degree of geo-indistinguishability $\varepsilon^{\prime }$ achieved by these mechanisms at each moment after the filtering attack. As shown in Figure 14d, the expected privacy strength is indicated by the dashed line $\sqrt{2}/\lambda$, and the results of DCLM, NonQCLM, and QCLM-Lowpass are close to it, demonstrating their effectiveness against the filtering attack. However, QCLM-Lowpass’s results are more consistent with expectations, while the other two show fluctuations due to the undesired transient response. In addition, CLM-Lowpass in NonQCLM may exacerbate variations of the filter parameter, resulting in greater fluctuations in the privacy strength.

In summary, QCLM-Lowpass has good performance both in terms of the stability of privacy protection for location series and the capability of resisting the filtering attack, and thus it is feasible to apply in continuous location data release.

### 5.4. Performance Evaluation

The actual performance of the privacy scheme was evaluated on real datasets, considering two main aspects: the privacy performance and the data availability.

1. For privacy performance, this paper focuses on the ability to resist correlation-based attacks, which was evaluated by comparing the change in privacy strength before and after the filtering attack.
2. For data availability, this paper evaluated the ability to balance privacy protection and data availability by comparing the data availability at the same level of privacy strength under the filtering attack.

These methods based on CLM in this paper were compared with the method based on a Markov model, called Markov-GRR [13]. Referring to the setup of that work, all trajectories within the second ring of Beijing (116°35′05″ E–116°45′59″ E, 39°87′36″ N–39°95′71″ N) were extracted from the GeoLife dataset to train the probability transfer matrix of the Markov model, i.e., the public matrix, and the region was divided into 200×200 grids, where each grid was approximately $44\times 44\;m^{2}$.

From the datasets with Δt=1 s and Δt=5 s in Section 5.1.2, we randomly selected 10 location series within the second ring of Beijing as the experimental dataset. Considering that the noise cannot be too large in practical applications, the scale of Laplace distribution was set as λ=20, 30, 40, 50, 60, the parameter of general random response in Markov-GRR was set as $\varepsilon_{GRR}=1.5,\;2,\;2.5,\;3,\;3.5,\;4$ with $\delta_{GRR}=0.01$ for the “δ-location set”, and the radius of the focused area was $r_{eff}=50$ m. The privacy strength of location dataset before and after the filtering attack, $E_{0.05},\;E_{0.05}^{\prime }$, were compared.

#### 5.4.1. Privacy Evaluation

**Figure 15:**
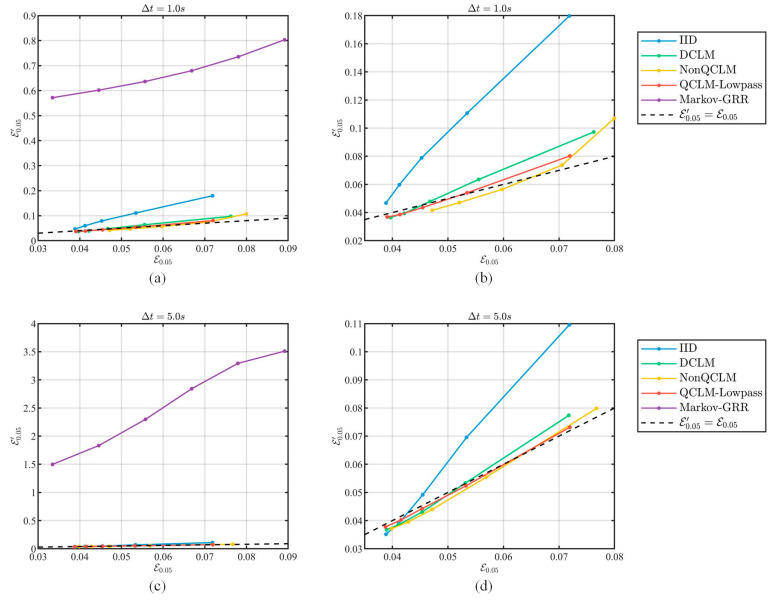
Actual privacy strength under the filtering attack. (**a**) Compared with Markov-GRR under Δt=1. (**b**) Compared without Markov-GRR under Δt=1. (**c**) Compared with Markov-GRR under Δt=5. (**d**) Compared without Markov-GRR under Δt=5.

Figure 15 shows the privacy strength achieved by different privacy schemes before and after the filtering attack, and the black dashed line $E_{0.05}^{\prime }=E_{0.05}$ indicates the case where the privacy strengths do not change. As can be seen from Figure 15a,c, the Markov-GRR is located above the curve of $E_{0.05}^{\prime }=E_{0.05}$, which implies that the actual privacy strengths are significantly lower than the expected results. This is because the scheme independently selects a perturbed location from the “δ-location set” at each moment, which is still an independent perturbation method, and thus struggles to resist correlation-based attacks.

In addition, as shown in Figure 15b,d, the results of DCLM, NonQCLM, and QCLM-Lowpass are close to the curve of $E_{0.05}^{\prime }=E_{0.05}$, which illustrates their capabilities against the filtering attack. Table 4 gives the relative changes in privacy strength, where QCLM-Lowpass exhibits lower changes in most cases, reflecting its better performance in privacy preservation.

#### 5.4.2. Usability Evaluation

**Figure 16:**
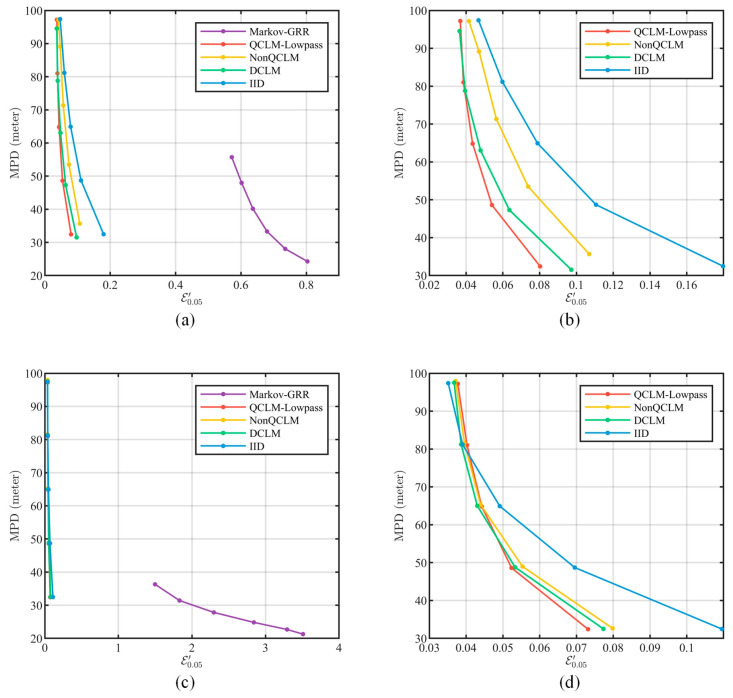
Mean perturbation distance and actual privacy strength. (**a**) Compared with Markov-GRR under Δt=1. (**b**) Compared without Markov-GRR under Δt=1. (**c**) Compared with Markov-GRR under Δt=5. (**d**) Compared without Markov-GRR under Δt=5.

First, MPD defined in Equation (54) was used to evaluate the generalized data availability. In Figure 16, as the actual privacy strength decreases, i.e., $E_{0.05}^{\prime }$ becomes larger, MPD decreases correspondingly, resulting in better data availability. The performance of Markov-GRR is affected by the grid granularity; Figure 16a,c present the results under the gridding scheme in this paper. As shown in Figure 16b,d, QCLM-Lowpass is essentially located below the rest of the schemes, which implies that QCLM-Lowpass induces less data availability loss at the same level of privacy strength. In contrast, DCLM and NonQCLM induce unwanted transient responses during filter adjustment, which leads to increased loss of data availability.

**Figure 17:**
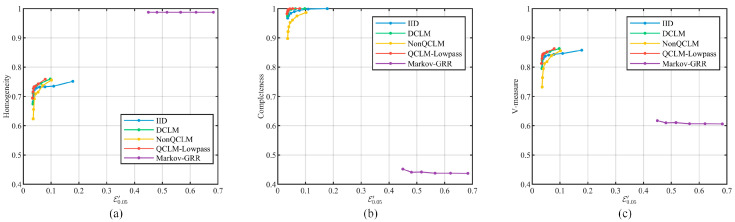
Evaluation of clustering results. (**a**) Homogeneity. (**b**) Completeness. (**c**) V-measure.

Furthermore, we clustered all location series within the second ring of Beijing using DBSCAN [27], and evaluated the data availability achieved by the privacy scheme using homogeneity, completeness, and V-measure [28], as shown in Figure 17. In Markov-GRR, the perturbed locations are scattered over the grid, which leads to an increase in the cluster number and worse performance in terms of completeness. In the CLM-based schemes, the results of QCLM-Lowpass are located above other schemes at the same privacy strength, indicating that it can achieve better data availability in clustering application.

To summarize the above experimental results, it can be obtained that all of DCLM, NonQCLM, and QCLM-Lowpass can effectively resist the correlation-based attack, but QCLM-Lowpass can achieve a better balance between the actual privacy protection performance and the data availability.

## 6. Discussion and Conclusions

In this paper, the correlation Laplace mechanism was applied to ensure differential privacy protection for continuous release of real-time location data. To solve the problem of nonstationary location data correlation estimation, this paper analyzed the stationarity of real location data series and found that more than 27% of the real location series have approximately stationary increments. Therefore, a location data correlation estimation method based on the location increment was proposed, which provides support for CLM to track the time-varying data correlation. In addition, by exploiting the lowpass spectral characteristic of the location data series, CLM-Lowpass was proposed, in which series-indistinguishability is approximated by constructing a noise series with the same lowpass characteristics. This method simplifies the calculation process of CLM filters. Finally, an adaptive adjustment scheme for CLM filters based on parameter quantization, QCLM-Lowpass, was proposed to suppress the unwanted transient response when tracking time-varying data correlation. Extensive experiments show that the privacy scheme based on QCLM-Lowpass can provide a better balance between the ability to resist correlation-based attacks and data usability.

Although QCLM-Lowpass is effective, there are still some aspects to be improved in the future. First, there are opportunities to further optimize the parameter settings, such as quasi-stationary thresholds, quantization scheme, etc. Second, this paper ignores the inter-correlation between the data in the X and Y directions, which can be exploited by attackers to launch attacks on privacy preservation, and needs to be investigated in subsequent work on privacy preservation of 2D location data series.

Future work would consider more personalized privacy preservation, i.e., allowing the user to specify the degree of series-indistinguishability between the original and noise series, thus enabling a trade-off between location privacy and motion state privacy.

## Figures and Tables

**Table 1:** The definition of major notations.

Variable	Definition
$l(t)={\left[x(t),y(t)\right]}^{T}$	a location data in *XOY* at time t
Δt	the time interval for data release
$t_{i}$	the time of *i*-th data release
$l(i)={\left[x(i),y(i)\right]}^{T}$	the simplified representation of $l(t_{i})$ in time discretization
L(I)=[X(I),Y(I)]	the location data series up to *I*-th data release
$R_{L}(i,i-\tau )$	the autocorrelation function matrix of L(I), $R_{L}(i,i-\tau )=E[l(i)l^{T}(i-\tau )]$
$n(i)={\left[n_{X}(i),n_{Y}(i)\right]}^{T}$	the perturbation noise added to l(i) at time $t_{i}$
$N(I)=[N_{X}(I),N_{Y}(I)]$	the noise series up to *I*-th data release
$\tilde{l}(i)={\left[\tilde{x}(i),\tilde{y}(i)\right]}^{T}$	the perturbed location data at time $t_{i}$
$\tilde{L}(I)=[\tilde{X}(I),\tilde{Y}(I)]$	the perturbed location data series up to *I*-th data release
$R_{\tilde{L}}(i,i-\tau )$	the autocorrelation function matrix of $\tilde{L}(I)$, $R_{\tilde{L}}(i,i-\tau )=E[\tilde{l}(i){\tilde{l}}^{T}(i-\tau )]$
$v(i)={\left[v_{X}(i),v_{Y}(i)\right]}^{T}$	the location increment at time $t_{i}$, v(i)=l(i)−l(i−1)
|v(i)|,φ(i)	the modulus |v(i)| and azimuth φ(i) of v(i)
V(I)	the location increment series up to *I*-th data release

**Table 2:** Main attributes and methods of the CLM object.

CLM		
Attributes		
Q,U	integer	the order of zeros and poles of the CLM filter
$B=[b_{1},\cdots ,b_{Q}]$	1×Q vector	$b_{q},q=1,\cdots ,Q$ are coefficients of the numerator of $H_{CLM}(z)$, where $b_{0}=1$
$A=[a_{1},\cdots ,a_{U}]$	1×U vector	$a_{u},u=1,\cdots ,U$ are coefficients of the denominator of $H_{CLM}(z)$, where $a_{0}=1$
κ,λ	real	κ: the gain coefficient; λ: the scale of Laplace distribution
$S=[s_{m,k}]$	4×Q matrix	the input state of four CLM filters, and each row corresponds to one filter
$S^{\prime }=[s_{m,k}^{\prime }]$	4×U matrix	the output state of four CLM filters and each row corresponds to one filter
$G={\left[g_{1},g_{2},g_{3},g_{4}\right]}^{T}$	4×1 vector	$g_{m}\sim N(0,1),m=1,\;2,\;3,\;4$ are the input of four CLM filters
$G^{\prime }={\left[g_{1}^{\prime },g_{2}^{\prime },g_{3}^{\prime },g_{4}^{\prime }\right]}^{T}$	4×1 vector	$g_{m}^{\prime },m=1,\;2,\;3,\;4$ are the output of four CLM filters
Methods		
Initialization(Q,U,B,A,κ,λ): Set Q,U,B,A,κ,λ; Set S: 4×Q zeros matrix, $S^{\prime }$: 4×U zeros matrix.		
$\left\{g_{1},g_{2},g_{3},g_{4}\}=GaussianGenerator(\;\right)$: Generate four i.i.d random numbers $g_{1},g_{2},g_{3},g_{4}\sim N(0,1)$.		
UpdateParam(B,A): Update B,A.		
UpdateGainCoeff(κ): Update κ.		
n=LaplaceGeneration( ): Generate the Laplace distributed noise n.		

**Table 3:** Main attributes and methods of the QCLM-Lowpass object.

QCLM-Lowpass		
Attributes		
Param	$QNum\times O_{CLM}$ matrix	the parameter vectors at different levels, defined in Section 4.3.2
GainCoeff	$QNum\times QNum\times (M_{T}+1)$ matrix	the gain coefficients in different level shifts, defined in Section 4.3.5
$K=[\kappa_{m}]$	$1\times (M_{T}+1)$ vector	the gain coefficient vector
ι	integer	the index of the gain coefficient vector
$\Psi =[\psi_{m}]$	$1\times (M_{\Psi }+1)$ vector	$\psi_{m},m=1,\cdots ,M_{\Psi }+1$ is the estimated level change, defined in Section 4.3.4
$\hat{L},L$	integer	$\hat{L},L$ denote the estimated and actual level of the CLM filter
Methods		
$Initialization(Param,GainCoeff,\Psi_{W},\hat{L},L,CLM)$: Set Param,GainCoeff; Set Ψ: $1\times (M_{\Psi }+1)$ zeros vector, and $\hat{L}=L=QNum$; Set K=GainCoeff(QNum,QNum), and the index ι=1; Initialize the member CLM;		
${\hat{L}}_{CLM}=Identify(\chi )$: Determine the estimated level ${\hat{L}}_{CLM}$ according to the feature parameter χ, defined in Section 4.3.4.		
n=Iteration(R): Adjust the CLM filter based on the autocorrelation function vector R, and generate the Laplace noise n.		

**Table 4:** Relative percentage difference in privacy strength under the filtering attack (%).

*λ*		20	30	40	50	60
Δt=1	IID	150.17	107.02	74.25	44.85	20.33
	DCLM	27.54	14.40	2.35	−6.41	−8.07
	NonQCLM	33.77	4.54	−5.52	−9.61	−11.88
	**QCLM-Lowpass**	**11.54**	**1.12**	**−4.18**	**−6.77**	**−5.63**
Δt=5	IID	52.47	30.37	8.14	−5.10	−9.52
	DCLM	7.80	0.38	−5.07	−6.30	−5.65
	NonQCLM	4.04	−2.64	−6.79	−7.70	−6.78
	**QCLM-Lowpass**	**1.67**	**−1.69**	**−2.42**	**−3.13**	**−2.58**

## Data Availability

Three publicly available datasets were analyzed in this study. Both datasets can be found here: [https://www.microsoft.com/en-us/research/publication/geolife-gps-trajectory-dataset-user-guide, https://www.microsoft.com/en-us/research/publication/t-drive-trajectory-data-sample, and https://www.openstreetmap.org/traces] (accessed on 17 July 2022).
